# Minor Prenylated Flavonoids from the Twigs of *Macarangaadenantha* and Their Cytotoxic Activity

**DOI:** 10.1007/s13659-015-0059-1

**Published:** 2015-04-10

**Authors:** Da-Song Yang, Shuang-Mei Wang, Wei-Bing Peng, Yong-Ping Yang, Ke-Chun Liu, Xiao-Li Li, Wei-Lie Xiao

**Affiliations:** Key Laboratory of Economic Plants and Biotechnology, Germplasm Bank of Wild Species in Southwest China, Institute of Tibetan Plateau Research at Kunming, Kunming Institute of Botany, Chinese Academy of Sciences, Kunming, 650201 People’s Republic of China; Biology Institute of Shandong Academy of Sciences, Jinan, 250014 People’s Republic of China; State Key Laboratory of Phytochemistry and Plant Resources in West China, Kunming Institute of Botany, Chinese Academy of Sciences, Kunming, 650201 People’s Republic of China

**Keywords:** *Macaranga adenantha*, Prenylated Flavonoids, Macadenanthins A–C, Cytotoxicity

## Abstract

Three new minor prenylated flavonoids, named macadenanthins A–C (**1**–**3**), together with three known ones (**4**–**6**), were isolated from the twigs of *Macaranga adenantha*. Their structures were elucidated on the basis of extensive spectroscopic analysis including NMR, UV and MS. The prenyl moieties in compounds **1**–**3** were further modified by cyclization and hydroxylation. The new compounds were tested for their cytotoxicity against four cancer cell lines (MCF-7, Hep G2, Hela and P388) and showed IC_50_ values in the range of 13.76–22.27 μM.

**Electronic supplementary material:**

The online version of this article (doi:10.1007/s13659-015-0059-1) contains supplementary material, which is available to authorized users.

## Introduction

Prenylated flavonoids are attracting great attention from the scientific community due to their structural uniqueness and remarkable biological activities [1]. The different prenylation position, various lengths of prenyl chain and further modifications on the prenyl moiety such as cyclization and hydroxylation resulted in the chemical diversity of the prenylated products, and which also made them exhibit promising biological activities. Prenylated flavonoids have a relatively narrow distribution in the plant kingdom and an overview of the literature indicated that prenylated flavonoids are the typical secondary metabolites of the genus *Macaranga* [2].

**Fig. 1:**
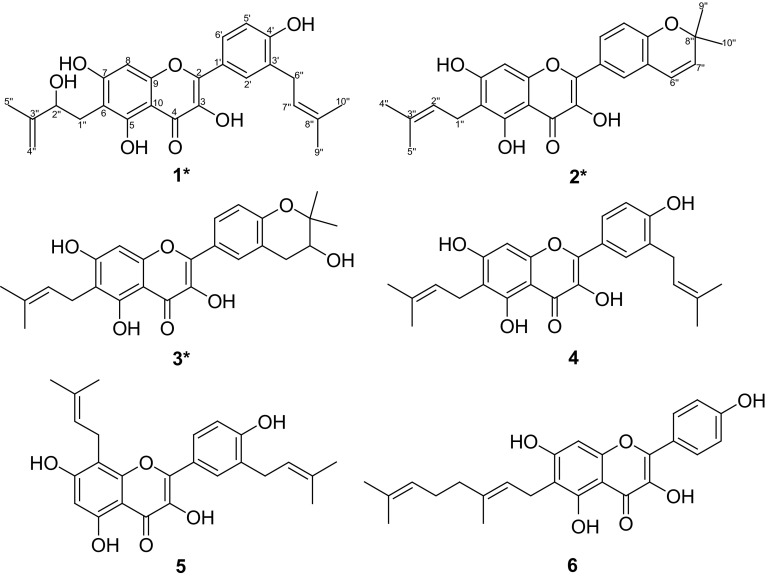
Structures of compounds **1**–**6**

*Macaranga adenantha* Gagnep (Euphorbiaceae) is an arbor distributed in the tropical rainforests, previous studies showed that it contained triterpenoids, steroids and phenolic compounds [3–5]. As part of our program to discover new anticancer prenylated aromatic products from the genus *Macaranga* [6–8], a phytochemical investigation on this plant led to the isolation and characterization of three minor new prenylated flavonoids, named macadenanthins A–C (**1**–**3**), along with three known ones, including glyasperin A (**4**) [9], broussoflavonol F (**5**) [10], and macarangin (**6**) [11]. The new compounds **1**–**3** were evaluated for their cytotoxicity against a small panel of cancer cell lines. Described herein are the isolation, structure elucidation and cytotoxicity of these compounds (Fig. 1).

## Results and Discussion

**Fig. 2:**
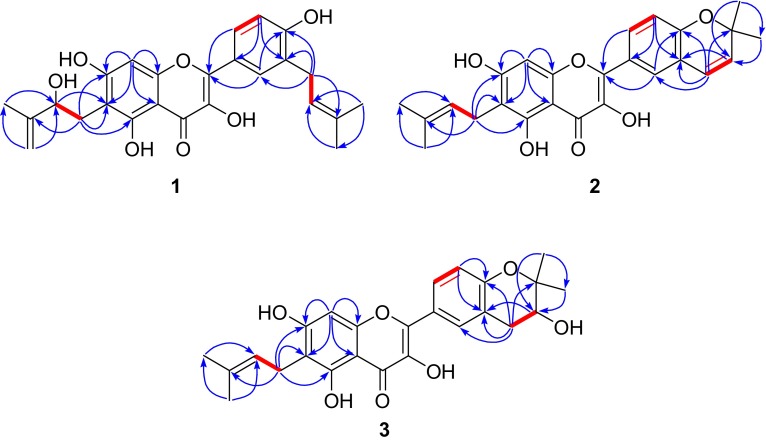
Selected HMBC () and COSY () correlations of compounds **1**–**3**

Macadenanthin A (**1**) was obtained as optically active yellow powder ($[\alpha]_{{\text{D}}}^{{21}}$ –1.9, *c* 0.12, MeOH). The molecular formula C_25_H_26_O_7_ was deduced from the HRESIMS (*m/z* 437.1609 [M–H]^−^), requiring 13° of unsaturation. The IR spectrum showed absorption bands for OH (3425 cm^−1^), carbonyl (1648 cm^−1^) and aromatic ring (1604 and 1488 cm^−1^) moieties. The UV spectrum showed absorption maxima at 270 and 380 nm, which indicated the presence of a flavonol skeleton [12]. Analysis of the ^1^H and ^13^C NMR (Table 1) data of **1** aided by HSQC revealed resonances for a hydrogen-bonded hydroxy group (*δ*_H_ 12.61, s); a 1,3,4-trisubstituted benzene ring (*δ*_H_ 7.00, 1H, d, *J* = 8.5 Hz; 7.98, 1H, dd, *J* = 8.5, 2.2 Hz; 8.07, 1H, d, *J* = 2.2 Hz); a 6-substituted kaempferol [*δ*_C_ 136.5 (s), 176.5 (s), 95.0 (d); *δ*_H_ 6.52 (1H, s)] [9, 10]; a prenyl [*δ*_C_ 29.1 (t), 123.2 (d), 133.1 (s), 25.9 (q), 17.9 (q); *δ*_H_ 3.40 (2H, d, *J* = 7.4 Hz), 5.38 (1H, t, *J* = 7.4 Hz), 1.74 (3H, s), 1.75 (3H, s)] [9] and a 2-hydroxy-3-methyl-3-butenyl group [*δ*_C_ 29.5 (t), 76.4 (d), 148.2 (s), 110.4 (t), 18.3 (q); *δ*_H_ 3.07 (1H, dd, *J* = 14.4, 3.6 Hz), 2.92 (1H, dd, *J* = 14.4, 7.9 Hz), 4.42 (1H, dd, *J* = 7.9, 3.6 Hz), 4.92 (1H, s), 4.76 (1H, s), 1.83 (3H, s)] [12]. These signals indicated that **1** should be a diprenylated kaempferol and the attachment of 2-hydroxy-3-methyl-3-butenyl and prenyl groups to C-6 and C-3′ were deduced from HMBC correlations of H-1″/C-5, C-6, C-7; H-2″/C-6; H-6″/C-2′, C-3′, C-4′ (Fig. 2). The COSY correlation of H-1″/H-2″ and the HMBC correlations of H-1″/C-3″; H-4″/C-2″, C-5″; and Me-5″/C-2″ revealed that the terminal double bond and oxygenated methine were belong to 6-prenyl moiety. Therefore, the structure of **1** was elucidated as 6-(2-hydroxy-3-methyl-3-butenyl)-3′-prenyl kaempferol.

**Table 1:** ^1^H and ^13^C NMR spectroscopic data for compounds **1**–**3** in acetone-*d*_6_

No	**1** ^a^		**2** ^b^		**3** ^a^	
	*δ*_C_	*δ*_H_ (*J* in Hz)	*δ*_C_	*δ*_H_ (*J* in Hz)	*δ*_C_	*δ*_H_ (*J* in Hz)
2	148.2 s		146.4 s		146.4 s	
3	136.5 s		137.0 s		136.7 s	
4	176.5 s		176.7 s		176.6 s	
5	159.5 s		158.7 s		158.8 s	
6	109.6 s		111.7 s		111.8 s	
7	164.2 s		162.9 s		163.6 s	
8	95.0 d	6.52, s	93.6 d	6.49, s	94.0 d	6.61, s
9	156.1 s		155.7 s		155.9 s	
10	103.8 s		104.0 s		103.7 s	
1′	123.4 s		124.8 s		115.8 s	
2′	130.3 d	8.07, d (2.2)	126.8 d	7.88, d (2.0)	130.4 d	7.98, br s
3′	129.0 s		122.0 s		121.4 s	
4′	157.8 s		155.5 s		155.7 s	
5′	115.8 d	7.00, d (8.5)	117.1 d	6.84, d (8.6)	117.7 d	6.87, d (9.3)
6′	128.0 d	7.98, dd (8.5, 2.2)	129.8 d	7.97, dd (8.6, 2.0)	127.9 d	7.98, br s
1″	29.5 t	3.07, dd (14.4, 3.6)	22.0 t	3.31, d (7.1)	22.0 t	3.35, d (7.2)
		2.92, dd (14.4, 7.9)				
2″	76.4 d	4.42, dd (7.9, 3.6)	123.2 d	5.23, t (7.1)	123.4 d	5.27, t (7.2)
3″	148.2 s		131.7 s		131.4 s	
4″	110.4 t	4.92, s	25.8 q	1.61, s	25.9 q	1.64, s
		4.76, s				
5″	18.3 q	1.83, s	17.8 q	1.74, s	17.9 q	1.77, s
6″	29.1 t	3.40, d (7.4)	122.6 d	6.45, d (9.9)	32.2 t	3.10, dd (16.5, 5.9)
						2.80, overlap
7″	123.2 d	5.38, t (7.4)	132.3 d	5.78, d (9.9)	69.5 d	3.86, dd (7.9, 5.9)
8″	133.1s		77.9 s		78.7 s	
9″	25.9 q	1.74, s	28.3 q	1.41, s	26.0 q	1.37, s
10″	17.9 q	1.75, s	28.3 q	1.41, s	21.1 q	1.29, s
5-OH		12.61, s				12.40, s

^a^Recorded at 600 MHz

^b^Recorded at 400 MHz

Macadenanthin B (**2**) possessed a molecular formula of C_25_H_24_O_6_ as deduced from its HREIMS (*m/z* 420.1576 [M]^+^), suggesting 14° of unsaturation. The IR spectrum of **2** indicated the characteristic bands of hydroxyl (3423 cm^−1^) and conjugated carbonyl (1648 cm^−1^) groups. Comparing the NMR spectral data (Table 1) of **2** with those of **1** revealed that it shared the same molecular-skeleton with **1** and the major differences between them could be rationalized to the signals corresponding to the 2-hydroxy-3-methyl-3-butenyl group in **1** was replaced by a 2,2-dimethylpyran ring [*δ*_C_ 122.6 (d), 132.3 (d), 77.9 (s), 28.3 × 2 (q); *δ*_H_ 6.45 (1H, d, *J* = 9.9 Hz), 5.78 (1H, d, *J* = 9.9 Hz), 1.41 (6H, s)] [13] in **2**, which was consistent with its molecular formula. This deduction was further supported by the COSY correlation of H-6″/H-7″ and the HMBC correlations of H-6″/C-8″; Me-9″/C-7″, C-10″; and Me-10″/C-7″ (Fig. 2). The observed HMBC correlations of H-1″/C-5, C-6, C-7; H-6″/C-2′, C-3′, C-4′; and H-7″/C-3′ indicated the prenyl and 2,2-dimethylpyran moieties were connected with C-6 and C-3′, respectively. As a result, the structure of **2** was determined as 6-prenyl-3′,4′-(2,2-dimethylpyrano) kaempferol.

Macadenanthin C (**3**) was obtained as optically active yellow powder ($[\alpha]_{{\text{D}}}^{{21}}$ –3.3, *c* 0.12, MeOH) and the molecular formula was deduced to be C_25_H_26_O_7_ based on its HREIMS (*m/z* 438.1675 [M]^+^), suggesting 13° of unsaturation. The UV spectrum showed the characteristic absorbances for a flavonol [*λ*_max_ (log *ε*) 270 (4.47) and 376 (4.27) nm] [12]. The ^1^H and ^13^C NMR data of **3** (Table 1) were similar with those of **2** and the differences between them could be rationalized to the 2,2-dimethylpyran ring in **2** were replaced by a 2,2-dimethyl-3-hydroxydihydropyrano ring [*δ*_C_ 32.2 (t), 69.5 (d), 78.7 (s), 26.0 (q), 21.1 (q); *δ*_H_ 3.10 (1H, dd, *J* = 16.5, 5.9 Hz), 2.80 (1H, overlap), 3.86 (1H, dd, *J* = 7.9, 5.9 Hz), 1.37 (3H, s), 1.29 (3H, s)] [14] in **3**. This deduction was confirmed by the COSY correlation of H-6″/H-7″ and the HMBC correlations of H-6″/C-8″; Me-9″/C-7″, C-10″; and Me-10″/C-7″ (Fig. 2). The observed HMBC correlations of H-1″/C-5, C-6, C-7; H-6″/C-2′, C-3′, C-4′; H-7″/C-3′ (Fig. 2) demonstrated that prenyl and 2,2-dimethyl-3-hydroxydihydropyrano groups were located at C-6 and C-3′, respectively. Therefore, the structure of **3** was determined as 6-prenyl-3′,4′-(2,2-dimethyl-3-hydroxydihydropyrano) kaempferol.

Since prenylated flavonoids are reported to have modest or strong anticancer activities [1, 2, 6], the cytotoxicity of new compounds **1**–**3** were evaluated against human breast adenocarcinoma (MCF-7), human hepatocellular (Hep G2), human cervical carcinoma (Hela), mouse leukemia (P388) cell lines by MTT method, with 5-FU used as a positive control [15]. The results showed that compounds **2** and **3** exhibit cytotoxicity against Hep G2 cell line with IC_50_ values of 13.76 and 22.27 μM (IC_50_ of 5-FU was 102.01 μM), respectively. Compound **2** exhibit cytotoxicity against Hela cell line with IC_50_ value of 16.18 μM (IC_50_ of 5-FU 106.47 μM).

## Experimental Section

### General Experimental Procedures

Optical rotations were measured on a JASCO P-1020 digital polarimeter. CD spectra were obtained on an automated circular dichroism spectrometer (Applied Photophysics). UV spectra were obtained using a Shimadzu UV-2401A spectrophotometer. IR spectra were obtained on a Bruker Tenor 27 spectrometer with KBr pellets. 1D and 2D NMR spectra were recorded on Bruker AM-400, DRX-500 or AV III-600 spectrometers with TMS as an internal standard. ESIMS were recorded using a Finnigan MAT 90 instrument and HRESIMS was performed on an API QSTAR time-of-flight spectrometer, HREIMS were recorded on a Waters AutoSpec Premier P776 instrument. Column chromatography was performed on Sephadex LH-20 (Amersham Biosciences, Piscataway, USA), silica gel (200–300 mesh, Qingdao Marine Chemical Ltd., Qingdao, China), RP-18 gel (LiChroprep, 40–63 μm; Merck, Darmstadt, Germany), and MCI gel CHP20P (75–150 μm, Mitsubishi Chemical Corporation, Tokyo, Japan). Semipreparative HPLC was performed on a Agilent 1200 (column: Zorbax SB-C18, 250 × 9.4 mm; DAD detector). Fractions were monitored by TLC, visualized by heating silica gel plates sprayed with 15 % H_2_SO_4_ in EtOH.

### Plant Material

The twigs of *M. adenantha* were collected from Malipo County of Yunnan province, P. R. China, in June 2013. A voucher specimen (Yangyp-20130619) was deposited in the Herbarium of Kunming Institute of Botany, Chinese Academy of Sciences, which was identified by one of the authors (Prof. Yong-Ping Yang).

### Extraction and Isolation

The dried and powdered twigs of *M. adenantha* (9.5 kg) were extracted three times with 90 % EtOH (25 L) for 24 h at room temperature and filtrated. The filtrate was concentrated and partitioned between H_2_O and EtOAc and then the EtOAc portion was decolorized on MCI gel (eluting with 95 % EtOH). The residue (380 g) was chromatographed on silica gel (CHCl_3_–MeOH 1:0 to 1:1) to afford fractions A–F. Fraction B was purified over a Sephadex LH–20 CC (CHCl_3_–MeOH 1:1) and then fractionated by RP–18 gel (MeOH–H_2_O 40 to 100 %) to provide subfractions B1–B4. B1 was subjected to silica gel CC (CHCl_3_–EtOAc 4:1) and further purified by Sephadex LH-20 CC (CHCl_3_–MeOH 1:1) to afford **6** (1.0 mg). B2 was subjected to silica gel CC eluting with CHCl_3_–EtOAc (3:1) and further purified by semipreparative HPLC (MeOH − H_2_O, 65:35) to afford **2** (2.5 mg), **4** (0.8 mg) and **5** (1.2 mg). B3 was repeatedly chromatographed on silica gel and Sephadex LH–20 CC, followed by preparative TLC with CHCl_3_–EtOAc (4:1) to furnish **1** (1.9 mg) and **3** (1.3 mg).

### Macadenanthin A (**1**)

Yellow powder; $[\alpha]_{{\text{D}}}^{{21}}$ –1.9 (*c* 0.12, MeOH); UV (MeOH) *λ*_max_ (log *ε*) 205 (4.70), 270 (4.34), 384 (4.13), 426 (4.14) nm; IR (KBr) *ν*_max_ 3425, 2969, 2924, 2855, 1648, 1625, 1604, 1562, 1488, 1367, 1318, 1279, 1191, 1091, 902, 820, 583 cm^−1^; ^1^H and ^13^C NMR data see Table 1; ESIMS *m/z* 437 [M–H]^−^, 875 [2M–H]^−^; HRESIMS *m/z* 437.1609 (calcd for C_25_H_25_O_7_ [M–H]^−^, 437.1600).

### Macadenanthin B (**2**)

Yellow powder; $[\alpha]_{{\text{D}}}^{{21}}$ –2.5 (*c* 0.34, MeOH); UV (MeOH) *λ*_max_ (log *ε*) 211 (4.50), 236 (4.41), 361 (3.98), 430 (4.21) nm; IR (KBr) *ν*_max_ 3423, 2974, 2924, 1648, 1624, 1602, 1564, 1483, 1362, 1320, 1278, 1195, 1157, 1135, 1091, 1042, 960, 807 cm^−1^; ^1^H and ^13^C NMR data see Table 1; ESIMS *m/z* 419 [M–H]^−^, 839 [2M–H]^−^; HREIMS *m/z* 420.1576 (calcd for C_25_H_24_O_6_ [M]^+^, 420.1573).

### Macadenanthin C (**3**)

Yellow powder; $[\alpha]_{{\text{D}}}^{{21}}$ –3.3 (*c* 0.12, MeOH); UV (MeOH) *λ*_max_ (log *ε*) 204 (4.83), 270 (4.47), 376 (4.27), 426 (4.21) nm; IR (KBr) *ν*_max_ 3428, 2925, 2855, 1648, 1625, 1612, 1485, 1367, 1321, 1268, 1190, 1155, 1090, 1044, 822, 604, 543 cm^−1^; ^1^H and ^13^C NMR data see Table 1; ESIMS *m/z* 437 [M–H]^−^; HREIMS *m/z* 438.1675 (calcd for C_25_H_26_O_7_ [M]^+^, 438.1679).

### Cytotoxicity Bioassays

Compounds **1**–**3** were tested for their cytotoxicity against human breast adenocarcinoma (MCF-7), human hepatocellular (Hep G2), human cervical carcinoma (Hela), mouse leukemia (P388) by the MTT method, and 5-FU was used as a positive control. Briefly, 100 μL cell suspension (1 × 10^5^ cells/mL) was seeded into 96-well microteter plates and cultured for 24 h before the compound was added. Then, different concentrations of the compounds were added to the plates, the cells were cultivated for 48 h, and 10 μL of MTT (5 mg/mL) was added to each well. After 4 h, the culture medium was removed and the formazan crystal was completely dissolved with 150 μL DMSO to each well by vigorously shaking the plate. Finally, formazan absorbance was assessed by a BioRad microplate reader at 570 nm.

## Electronic supplementary material

Supplementary material 1 (PDF 2534 kb)
