# Supplementary material for: Minor Prenylated Flavonoids from the Twigs of Macarangaadenantha and Their Cytotoxic Activity
Source: Nat Prod Bioprospect. 2015 Apr 10;5(2):105–9. doi: 10.1007/s13659-015-0059-1 (PMC4402582; doi:10.1007/s13659-015-0059-1)
Supplement: Supplementary file 1 — Supplementary material 1 (PDF 2534 kb) [file 13659_2015_59_MOESM1_ESM.pdf]

Supporting Information for

Minor Prenylated Flavonoids from the Twigs of *Macaranga adenantha* and Their Cytotoxic Activity

Da-Song Yang,[a,†] Shuang-Mei Wang,[a,†] Wei-Bing Peng,[b] Yong-Ping Yang,[a] Ke-Chun Liu,[b] Xiao-Li Li,[a,*] Wei-Lie Xiao,[c,*]

[a] Key Laboratory of Economic Plants and Biotechnology; Germplasm Bank of Wild Species in Southwest China; Institute of Tibetan Plateau Research at Kunming, Kunming Institute of Botany, Chinese Academy of Sciences, Kunming 650201, P. R. China

[b] Biology Institute of Shandong Academy of Sciences, Jinan 250014, P. R. China

[c] State Key Laboratory of Phytochemistry and Plant Resources in West China, Kunming Institute of Botany, Chinese Academy of Sciences, Kunming 650201, P. R. China.

* Corresponding author. Tel/fax: (86) 871-65223231. Email: *li_xiaoli11@mail.kib.ac.cn* and *xwl@mail.kib.ac.cn*

† These authors contributed equally to this work.

Contents of Supporting Information

No.	Contents	Pages
1	Figure S1. Structures of compounds **1–6**	4
2	Figure S2. ^{1}H NMR spectrum of Macadenanthin A (**1**)	5
3	Figure S3. ^{13}C NMR spectrum of Macadenanthin A (**1**)	6
4	Figure S4. HSQC spectrum of Macadenanthin A (**1**)	7
5	Figure S5. HMBC spectrum of Macadenanthin A (**1**)	8
6	Figure S6. ^{1}H–^{1}H COSY spectrum of Macadenanthin A (**1**)	9
7	Figure S7. ROESY spectrum of Macadenanthin A (**1**)	10
8	Figure S8. ESIMS of Macadenanthin A (**1**)	11
9	Figure S9. HRESIMS of Macadenanthin A (**1**)	12
10	Figure S10. IR spectrum of Macadenanthin A (**1**)	13
11	Figure S11. UV spectrum of Macadenanthin A (**1**)	14
12	Figure S12. CD spectrum of Macadenanthin A (**1**)	15
13	Figure S13. ORD spectrum of Macadenanthin A (**1**)	16
14	Figure S14. ^{1}H NMR spectrum of Macadenanthin B (**2**)	17
15	Figure S15. ^{13}C NMR spectrum of Macadenanthin B (**2**)	18
16	Figure S16. HSQC spectrum of Macadenanthin B (**2**)	19
17	Figure S17. HMBC spectrum of Macadenanthin B (**2**)	20
18	Figure S18. ^{1}H–^{1}H COSY spectrum of Macadenanthin B (**2**)	21
19	Figure S19. ROESY spectrum of Macadenanthin B (**2**)	22
20	Figure S20. ESIMS of Macadenanthin B (**2**)	23
21	Figure S21. HREIMS of Macadenanthin B (**2**)	24
22	Figure S22. IR spectrum of Macadenanthin B (**2**)	25
23	Figure S23. UV spectrum of Macadenanthin B (**2**)	26
24	Figure S24. ORD spectrum of Macadenanthin B (**2**)	27
25	Figure S25. ^{1}H NMR spectrum of Macadenanthin C (**3**)	28
26	Figure S26. ^{13}C NMR spectrum of Macadenanthin C (**3**)	29
27	Figure S27. HSQC spectrum of Macadenanthin C (**3**)	30
28	Figure S28. HMBC spectrum of Macadenanthin C (**3**)	31
29	Figure S29. ^{1}H–^{1}H COSY spectrum of Macadenanthin C (**3**)	32
30	Figure S30. ROESY spectrum of Macadenanthin C (**3**)	33

31	Figure S31. ESIMS of Macadenanthin C (**3**)	34
32	Figure S32. HREIMS of Macadenanthin C (**3**)	35
33	Figure S33. IR spectrum of Macadenanthin C (**3**)	36
34	Figure S34. UV spectrum of Macadenanthin C (**3**)	37
35	Figure S35. CD spectrum of Macadenanthin C (**3**)	38
36	Figure S36. ORD spectrum of Macadenanthin C (**3**)	39

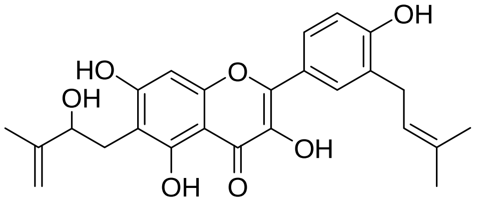

1*

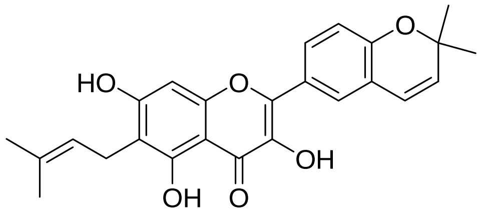

2*

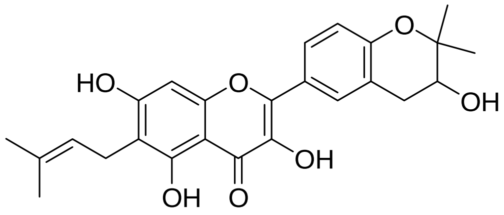

3*

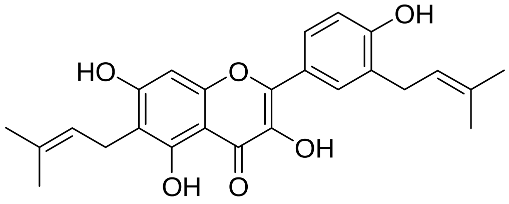

4

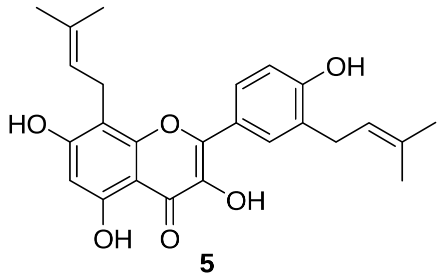

5

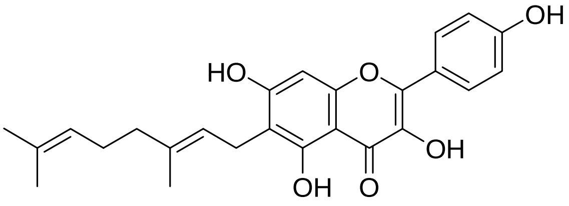

6

Figure S1. Structures of compounds **1–6**

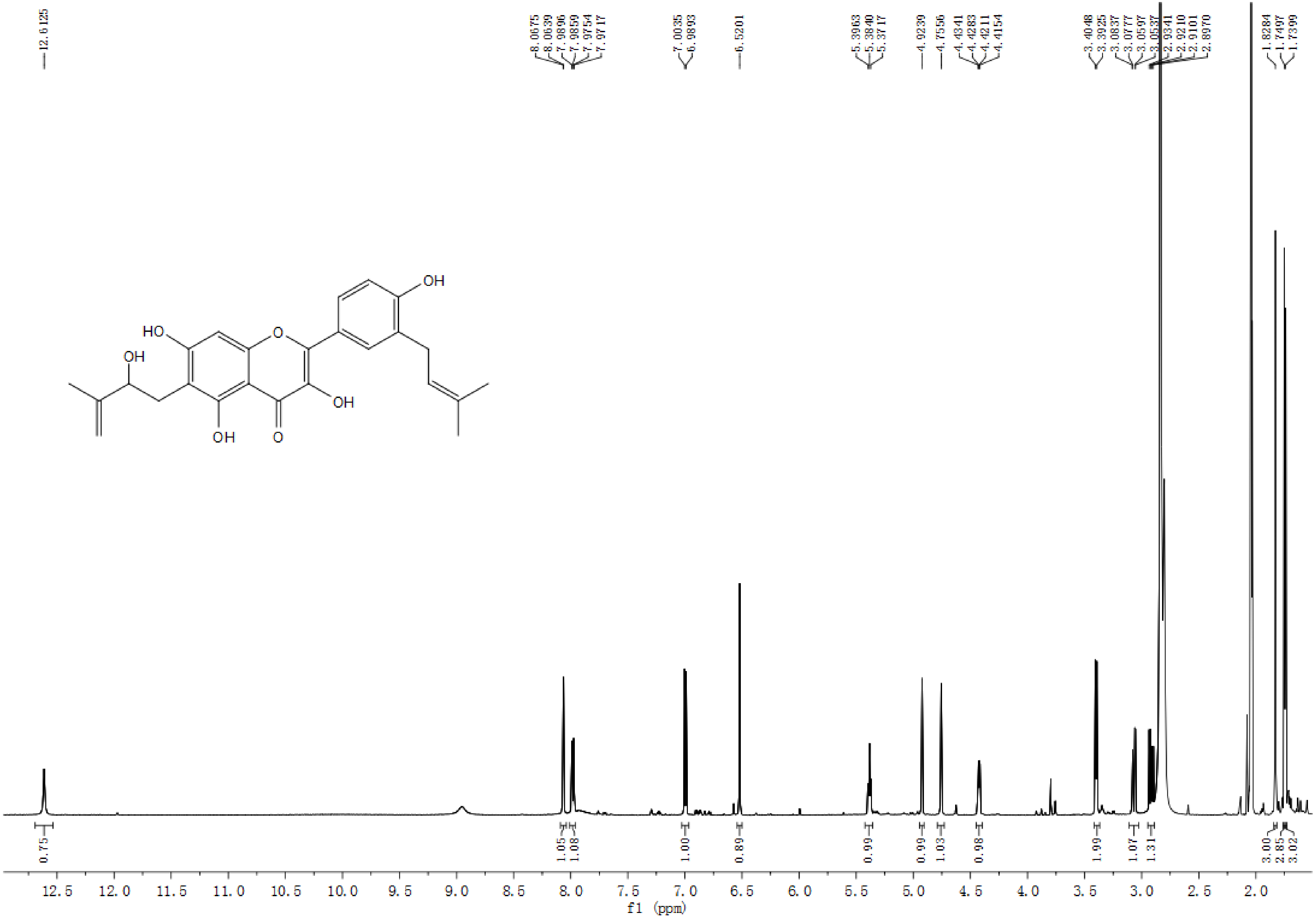

Figure S2. ^{1}H NMR spectrum of Macadenanthin A (**1**)

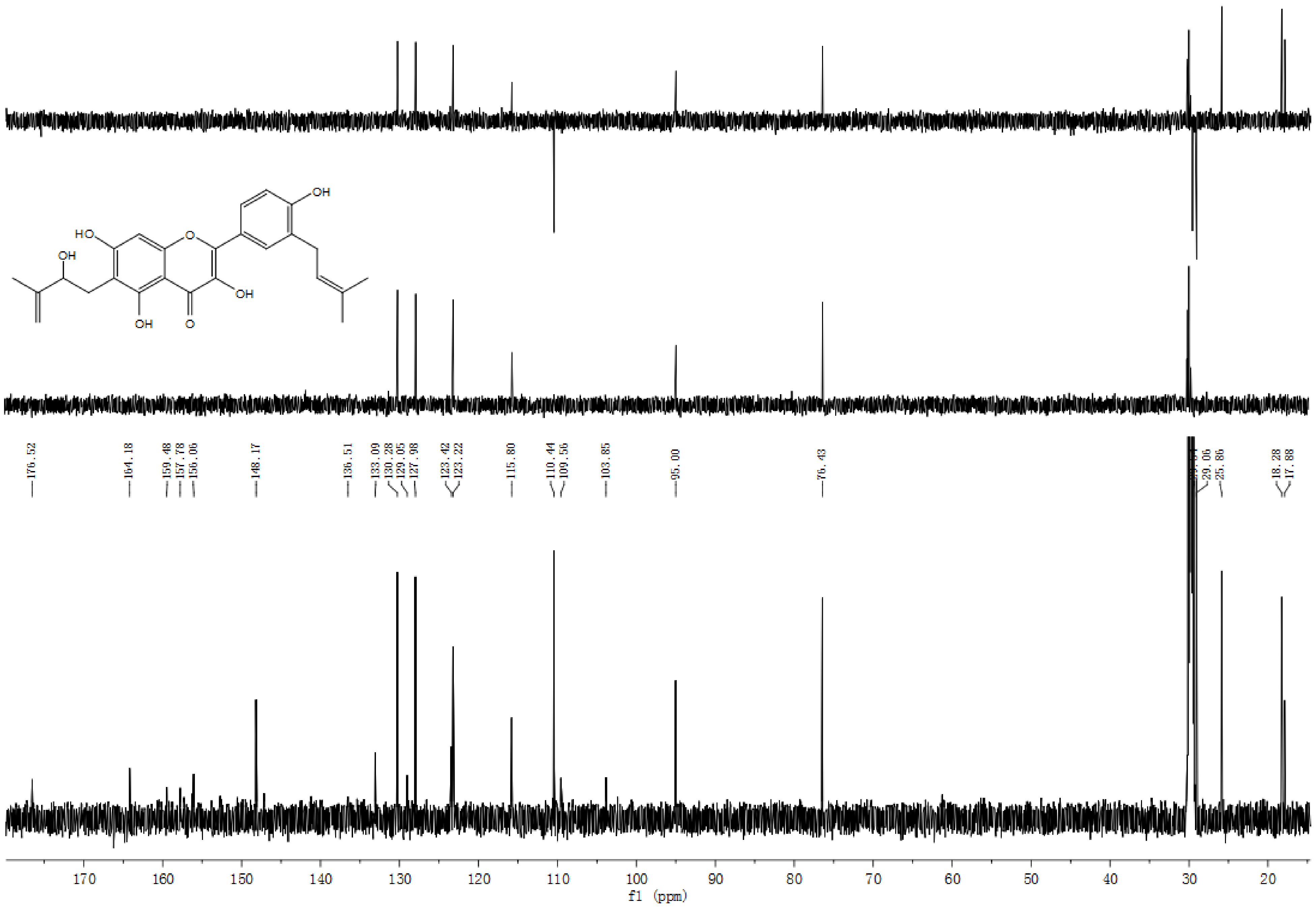

Figure S3. ^{13}C NMR spectrum of Macadenanthin A (**1**)

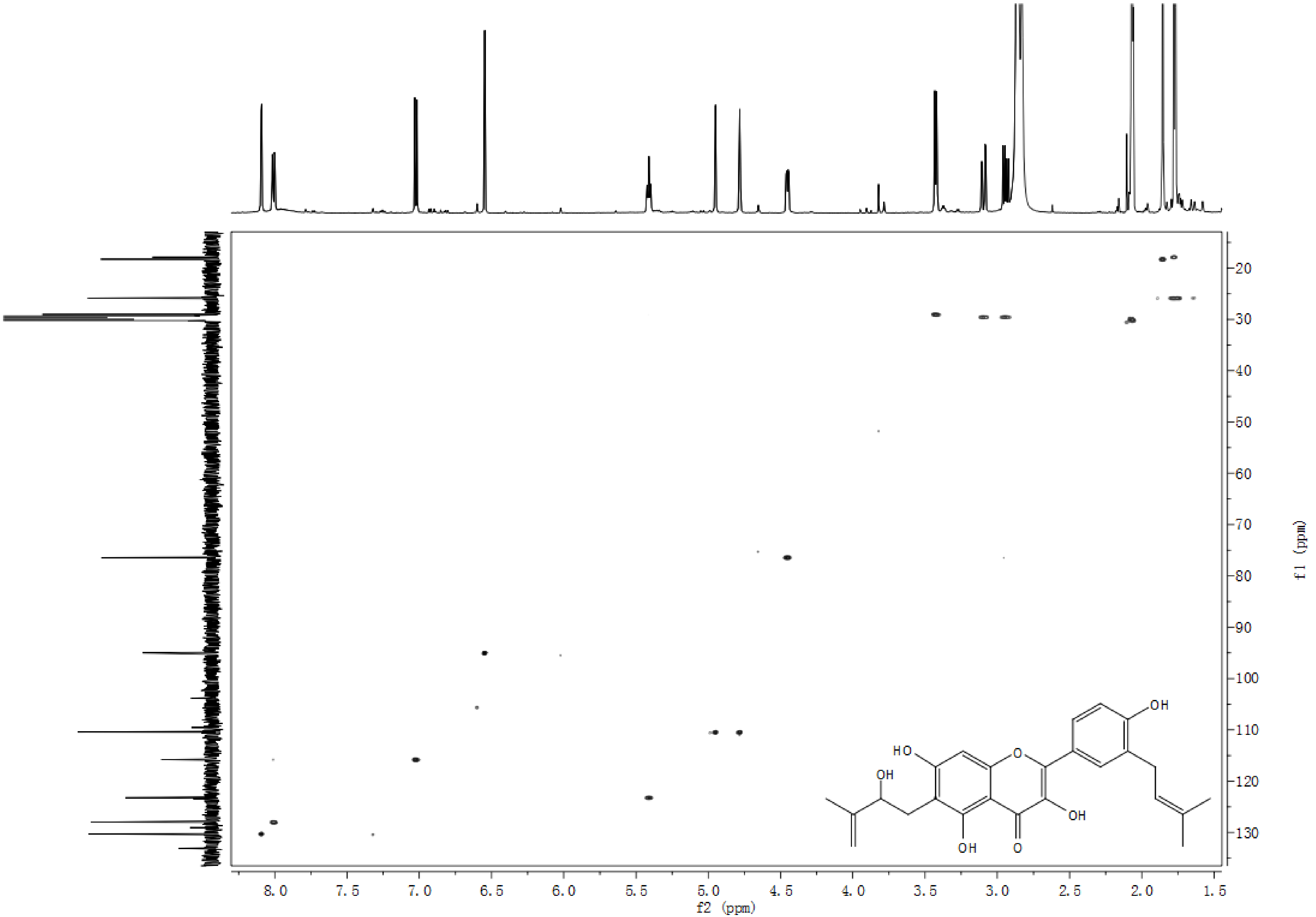

Figure S4. HSQC spectrum of Macadenanthin A (**1**)

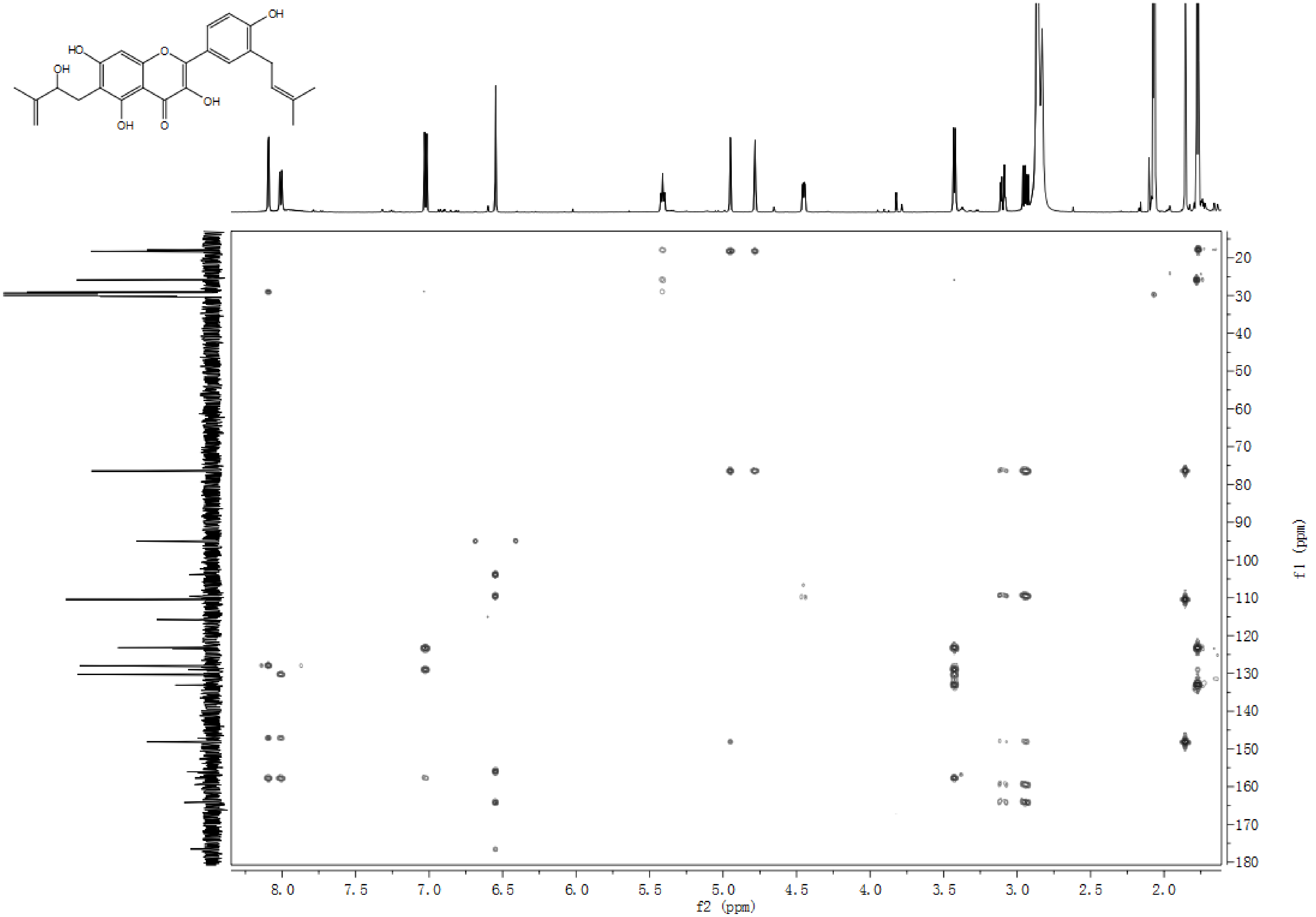

Figure S5. HMBC spectrum of Macadenanthin A (**1**)

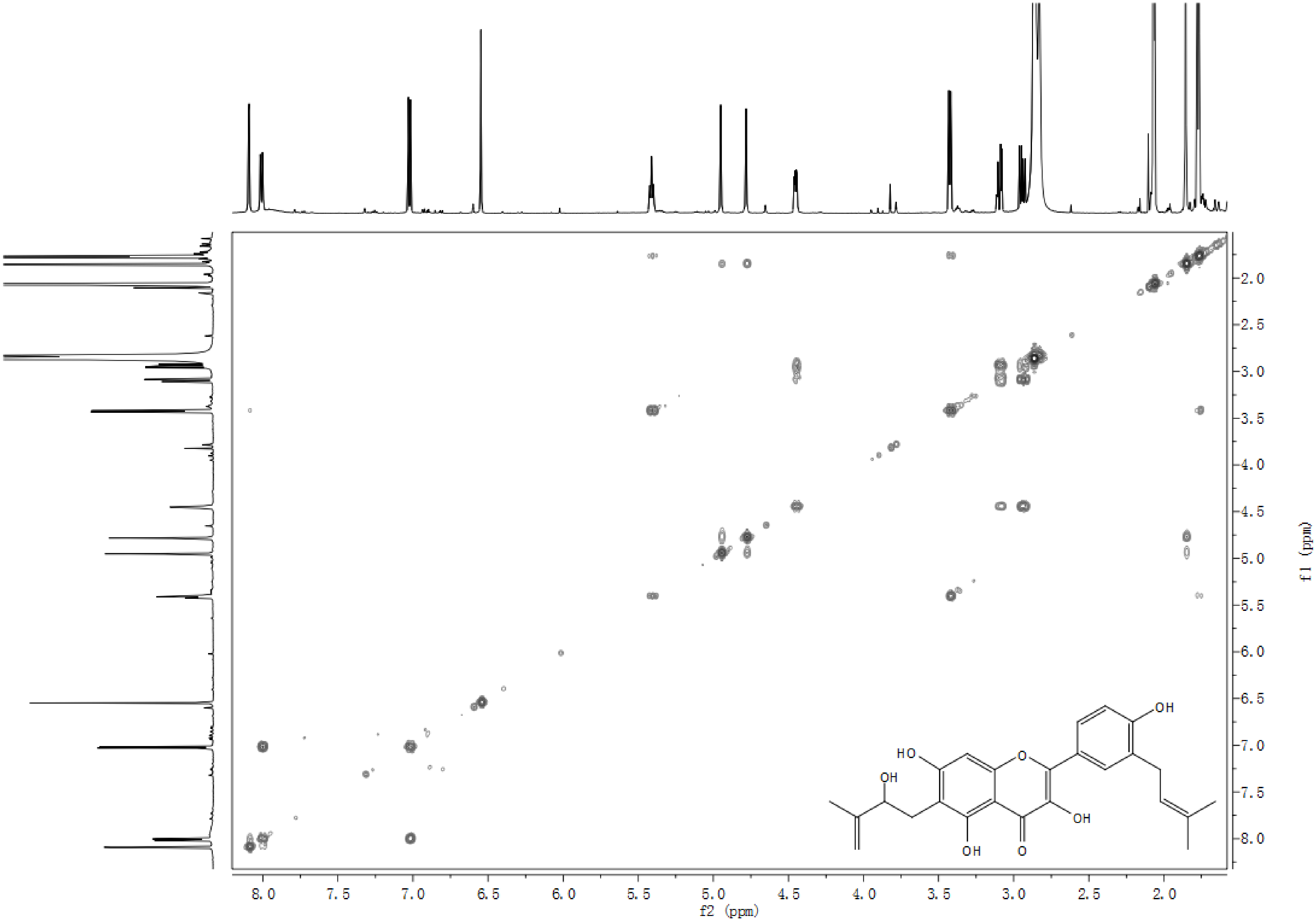

Figure S6. ^{1}H–^{1}H COSY spectrum of Macadenanthin A (**1**)

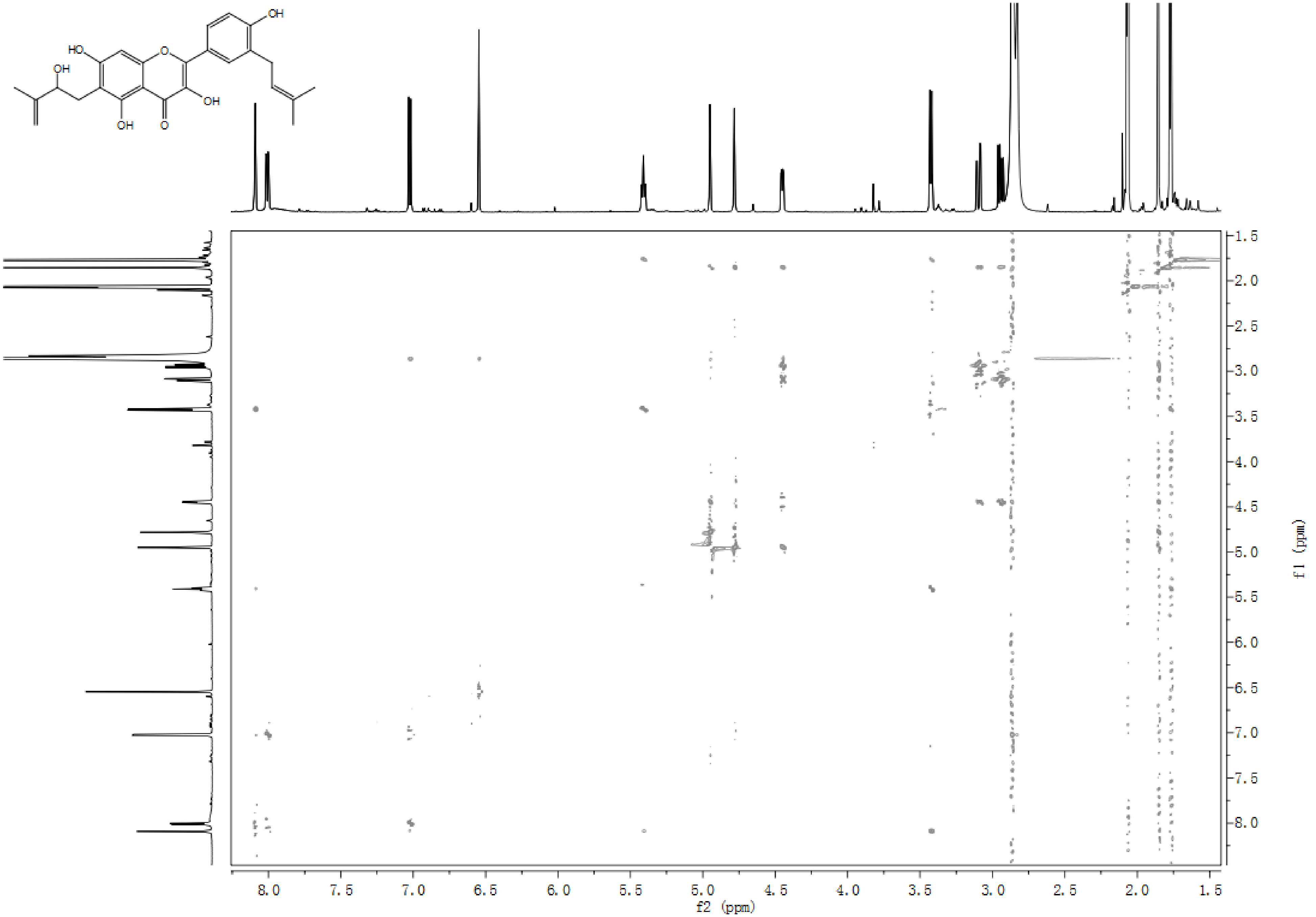

Figure S7. ROESY spectrum of Macadenanthin A (**1**)

Acquisition Parameter

Ion Source Type	ESI	Ion Polarity	Negative	Alternating Ion Polarity	off
Mass Range Mode	Ultra Scan	Scan Begin	100 m/z	Scan End	1200 m/z
Capillary Exit	-10.0 Volt	Skimmer	-40.0 Volt	Trap Drive	30.0
Accumulation Time	100000 µs	Averages	5 Spectra	Auto MS/MS	off

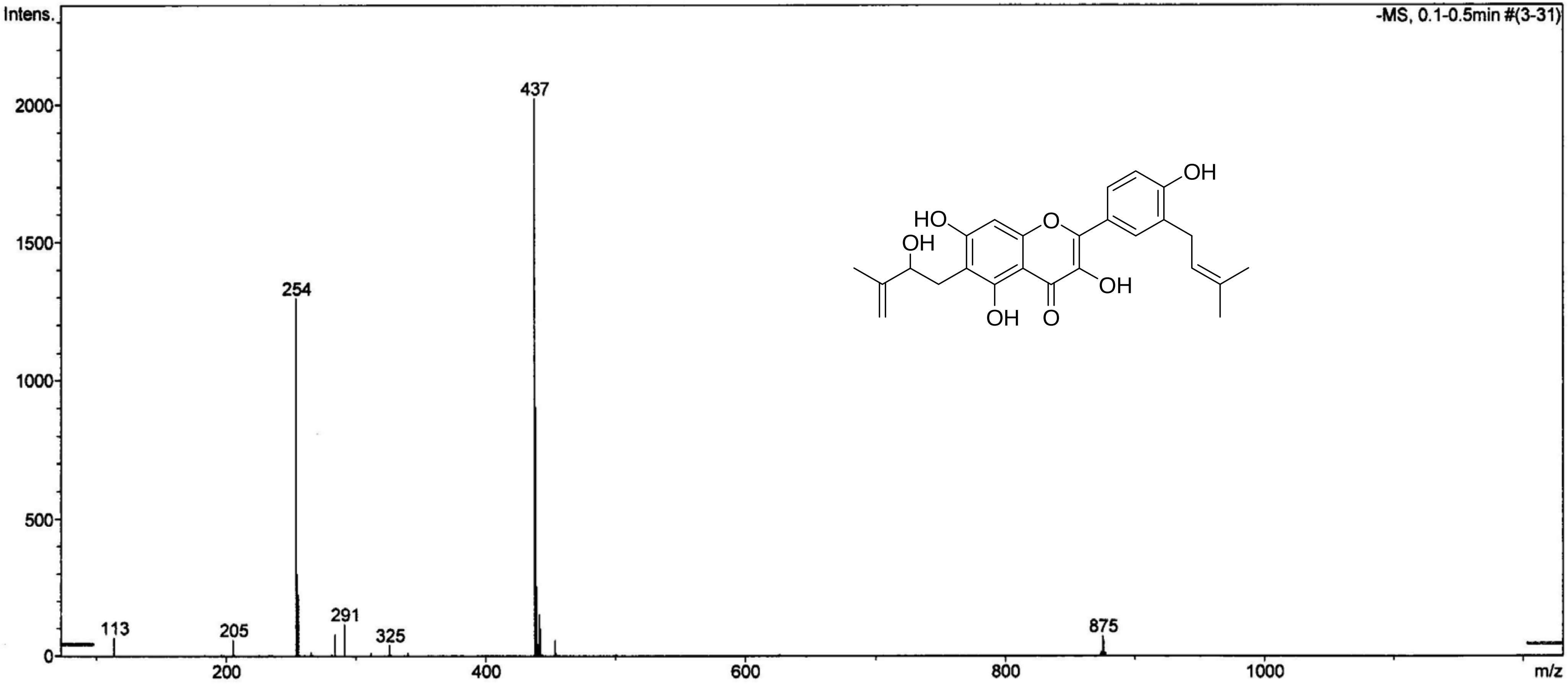

Figure S8. ESIMS of Macadenanthin (**1**)

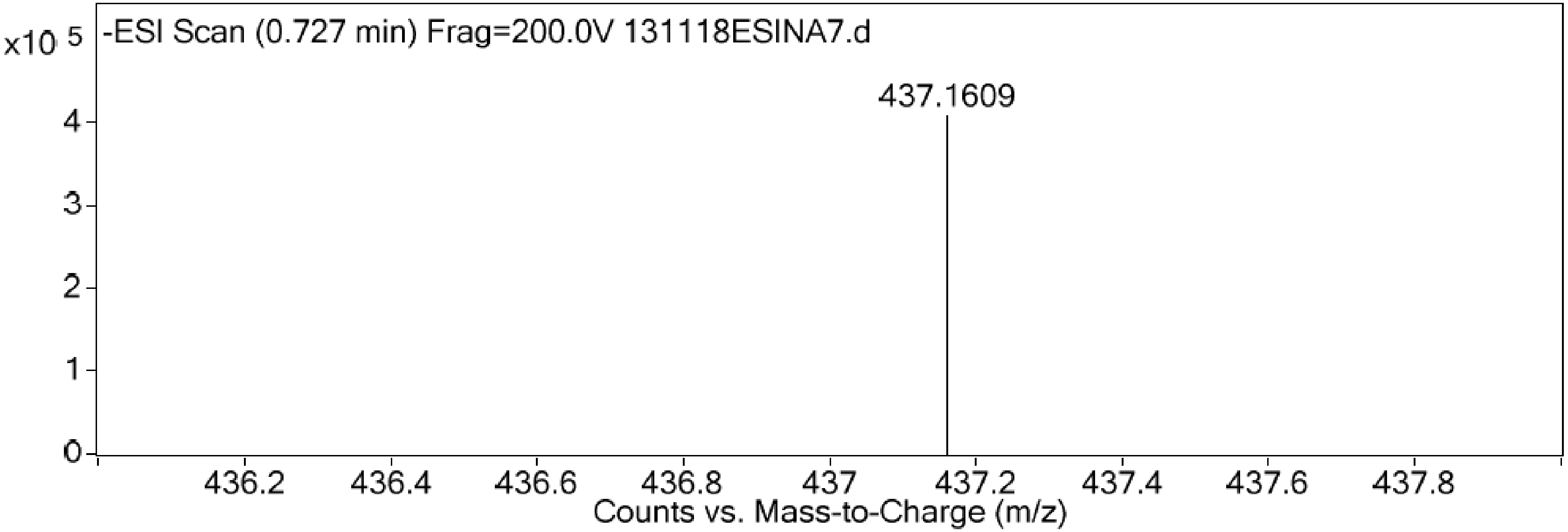

Formula Calculator Element Limits

Element	Min	Max
C	0	200
H	0	400
O	6	8

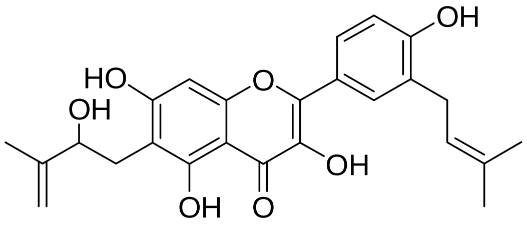

Formula Calculator Results

Formula	CalculatedMass	CalculatedMz	Mz	Diff. (mDa)	Diff. (ppm)	DBE
C25 H25 O7	437.1600	437.1606	437.1609	-0.4	-0.8	13.5000

Figure S9. HRESIMS of Macadenanthin A (**1**)

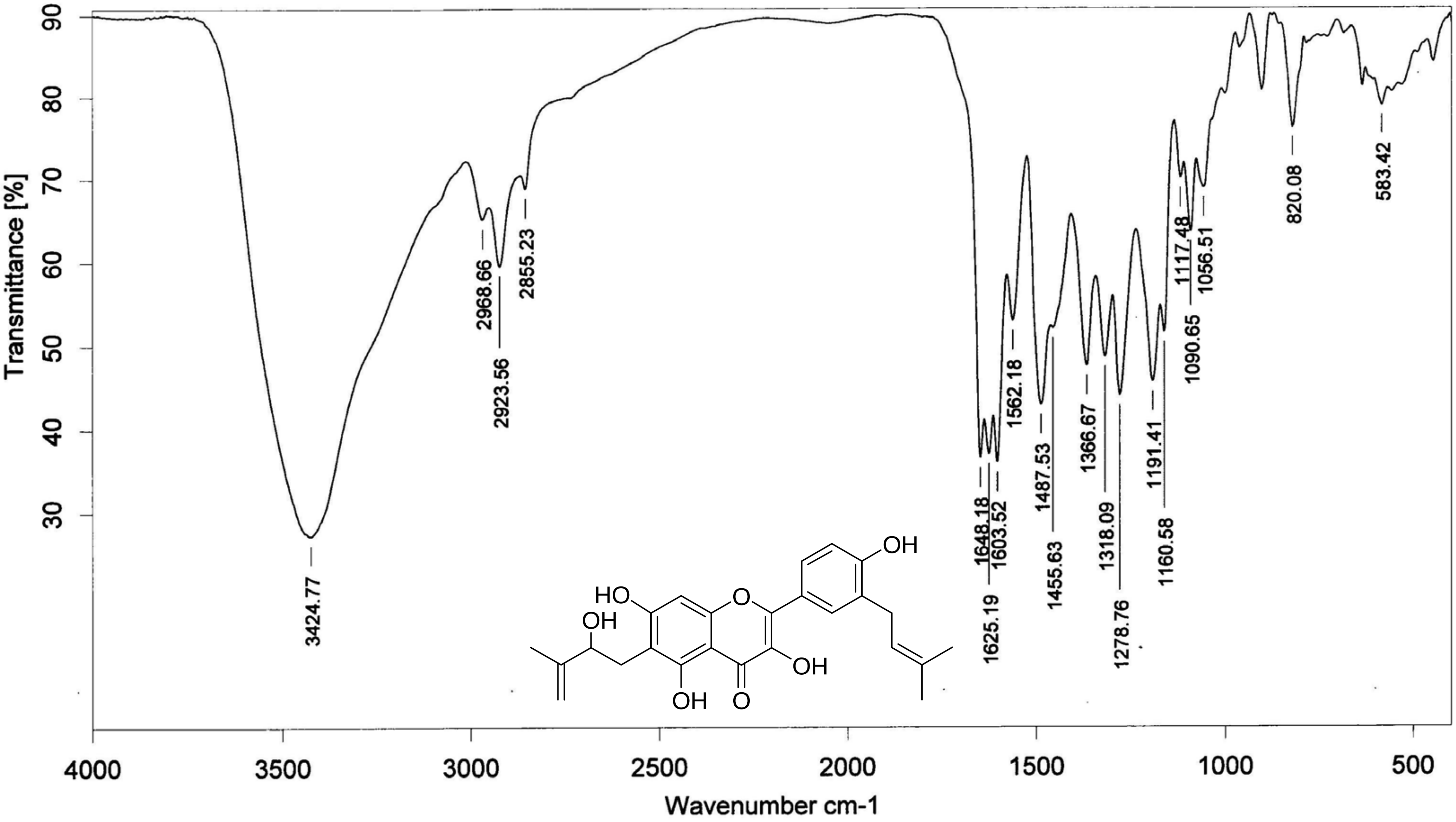

Figure S10. IR spectrum of Macadenanthin A (**1**)

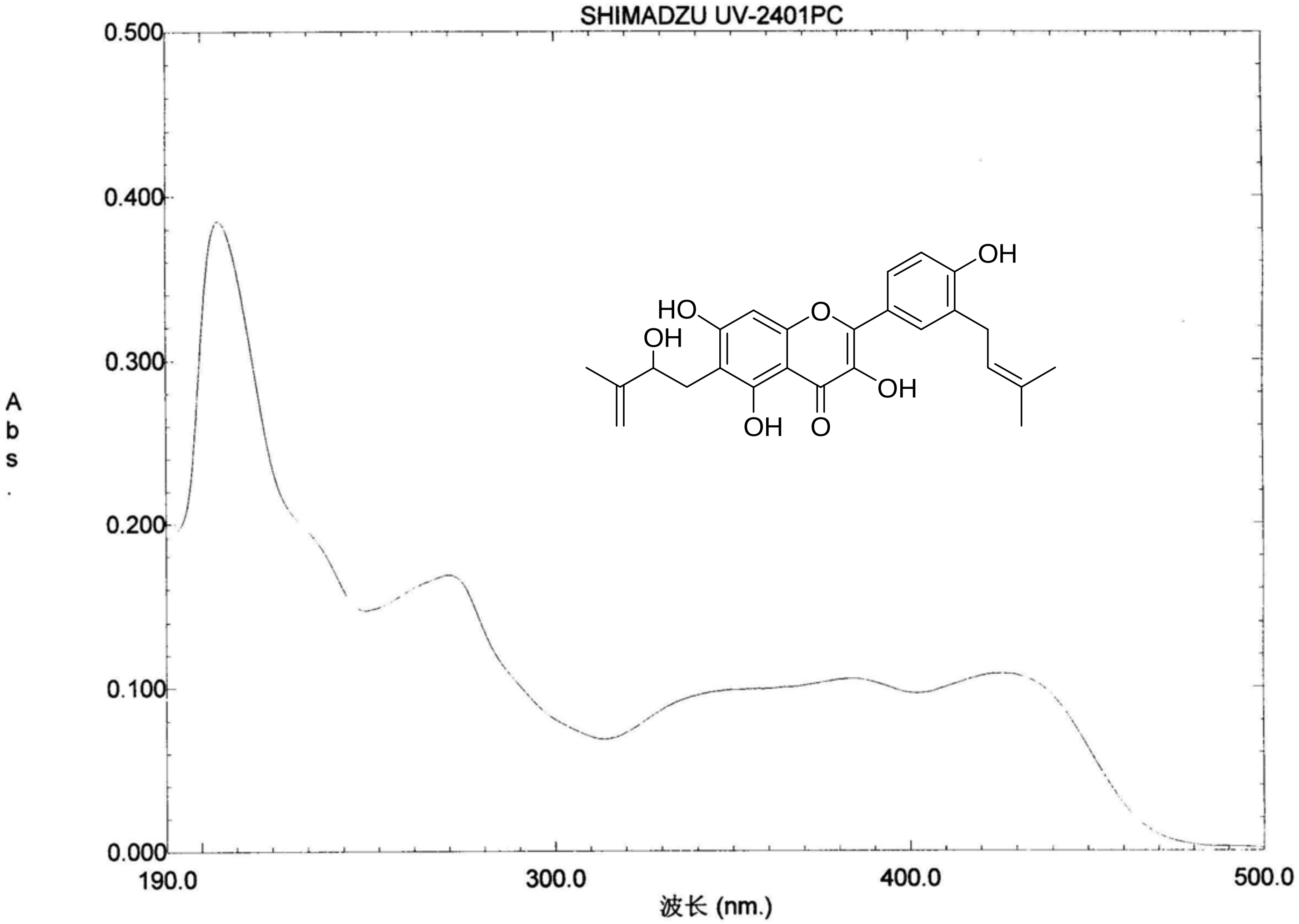

文件名: SMAW-5A

SMAW-5A

样品浓度：0.0034毫克/毫升
溶剂：甲醇

创建于: 16:40 13-11-22
数据: 原始

测量模式: Abs.
扫描速度: 中速
狭缝: 5.0
采样间隔: 0.5

否.	波长 (nm.)	Abs.
1	426.50	0.1080
2	384.00	0.1051
3	270.00	0.1682
4	205.00	0.3848

Figure S11. UV spectrum of Macadenanthin A (**1**)

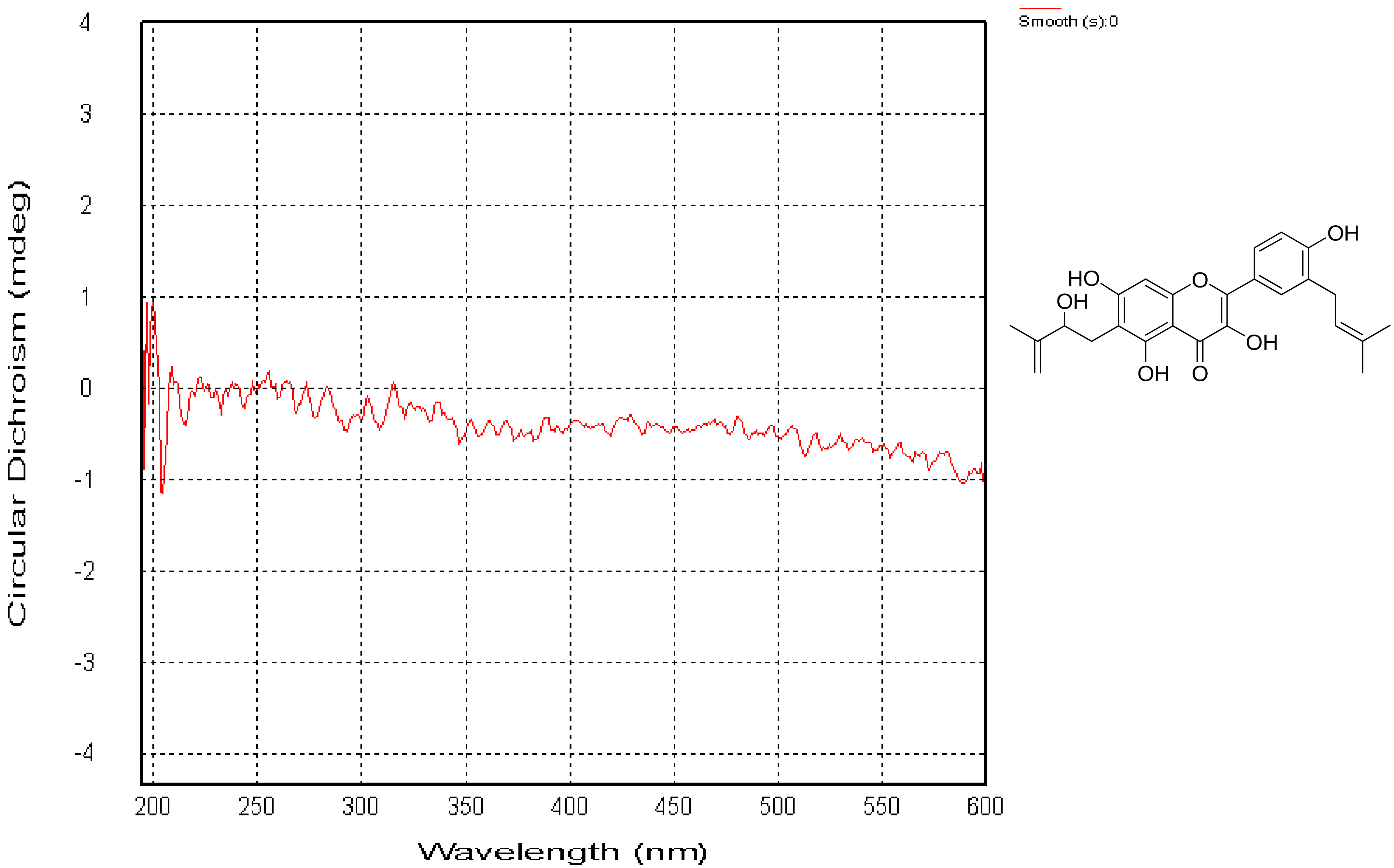

Figure S12. CD spectrum of Macadenanthin A (**1**)

Optical rotation measurement

Model : P-1020 (A060460638)

No.	Sample	Mode	Data	Monitor<br>Blank	Temp.<br>Cell<br>Temp Point	Date<br>Comment<br>Sample Name	Light<br>Filter<br>Operator	Cycle Time<br>Integ Time
No.1	11 (1/3)	Sp.Rot	-3.2520	-0.0004<br>0.0000	20.7<br>10.00<br>Cell	Fri Nov 22 14:36:11 2013<br>0.00123g/mL MeOH<br>SMAW-5A	Na<br>589nm	2 sec<br>10 sec
No.2	11 (2/3)	Sp.Rot	-1.6260	-0.0002<br>0.0000	20.8<br>10.00<br>Cell	Fri Nov 22 14:36:24 2013<br>0.00123g/mL MeOH<br>SMAW-5A	Na<br>589nm	2 sec<br>10 sec
No.3	11 (3/3)	Sp.Rot	-0.8130	-0.0001<br>0.0000	20.8<br>10.00<br>Cell	Fri Nov 22 14:36:37 2013<br>0.00123g/mL MeOH<br>SMAW-5A	Na<br>589nm	2 sec<br>10 sec

−1.8970°

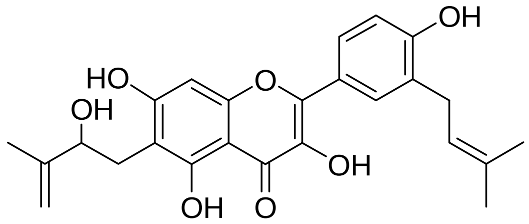

Figure S13. ORD spectrum of Macadenanthin A (**1**)

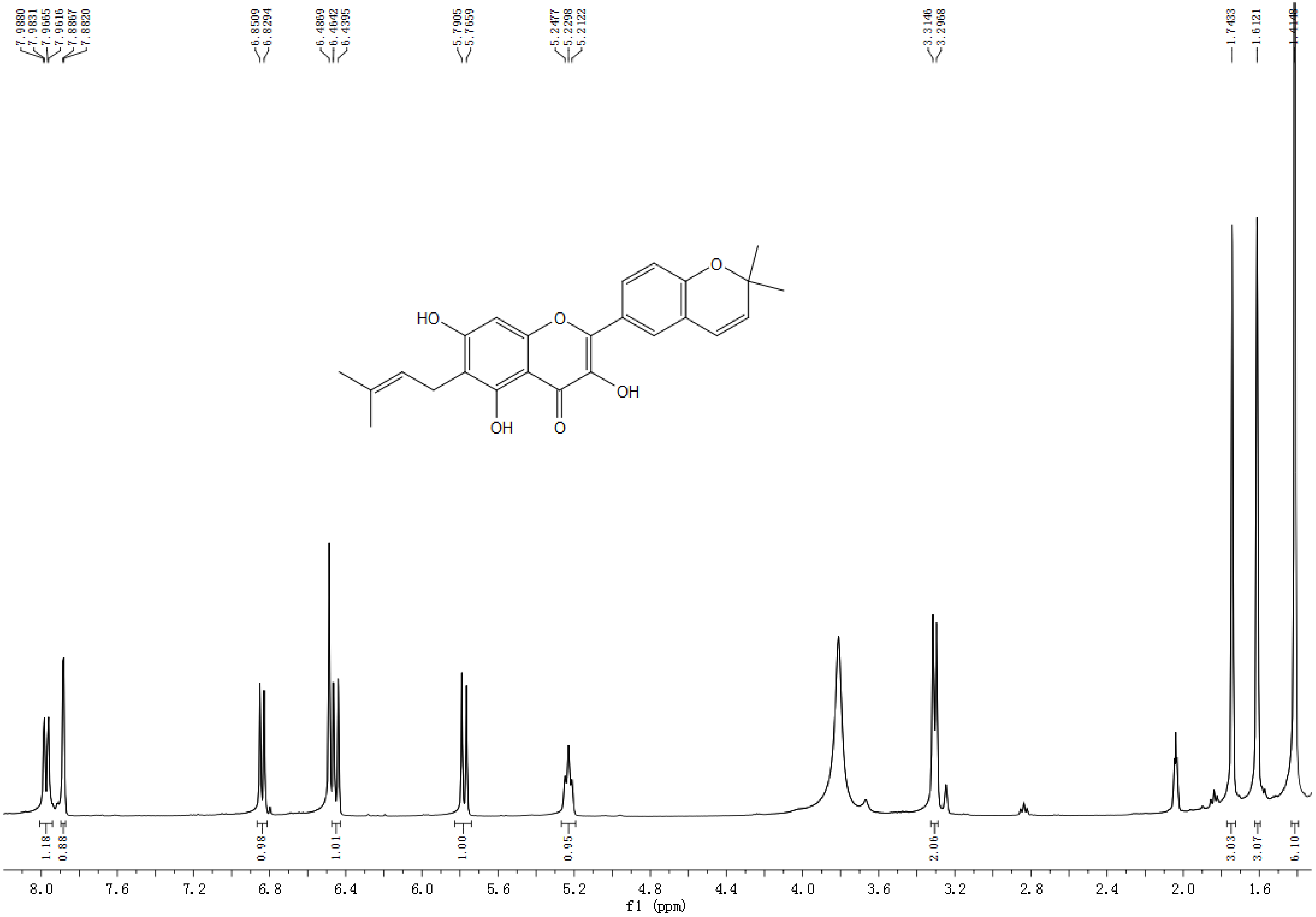

Figure S14. ^{1}H NMR spectrum of Macadenanthin B (**2**)

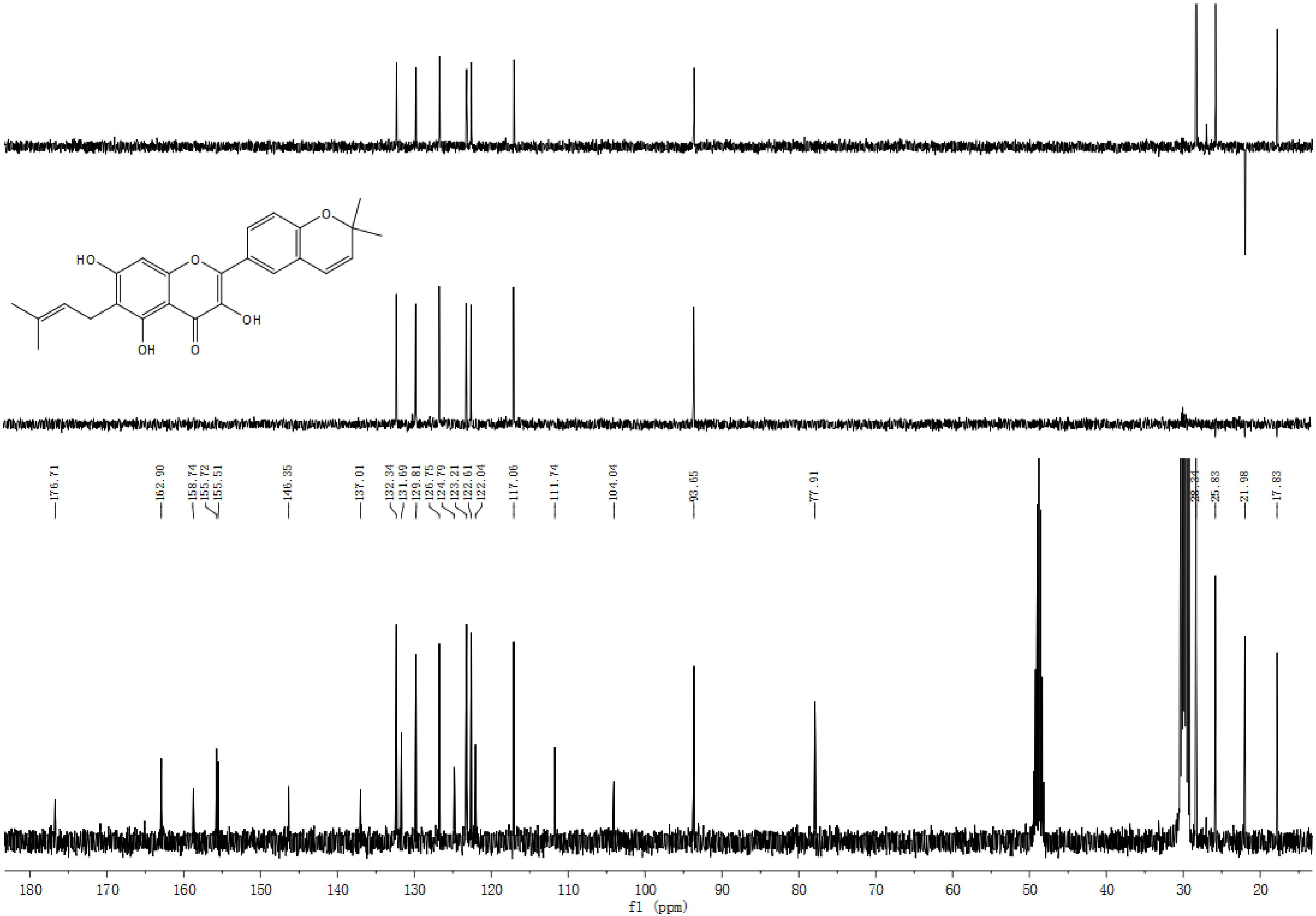

Figure S15. ^{13}C NMR spectrum of Macadenanthin B (**2**)

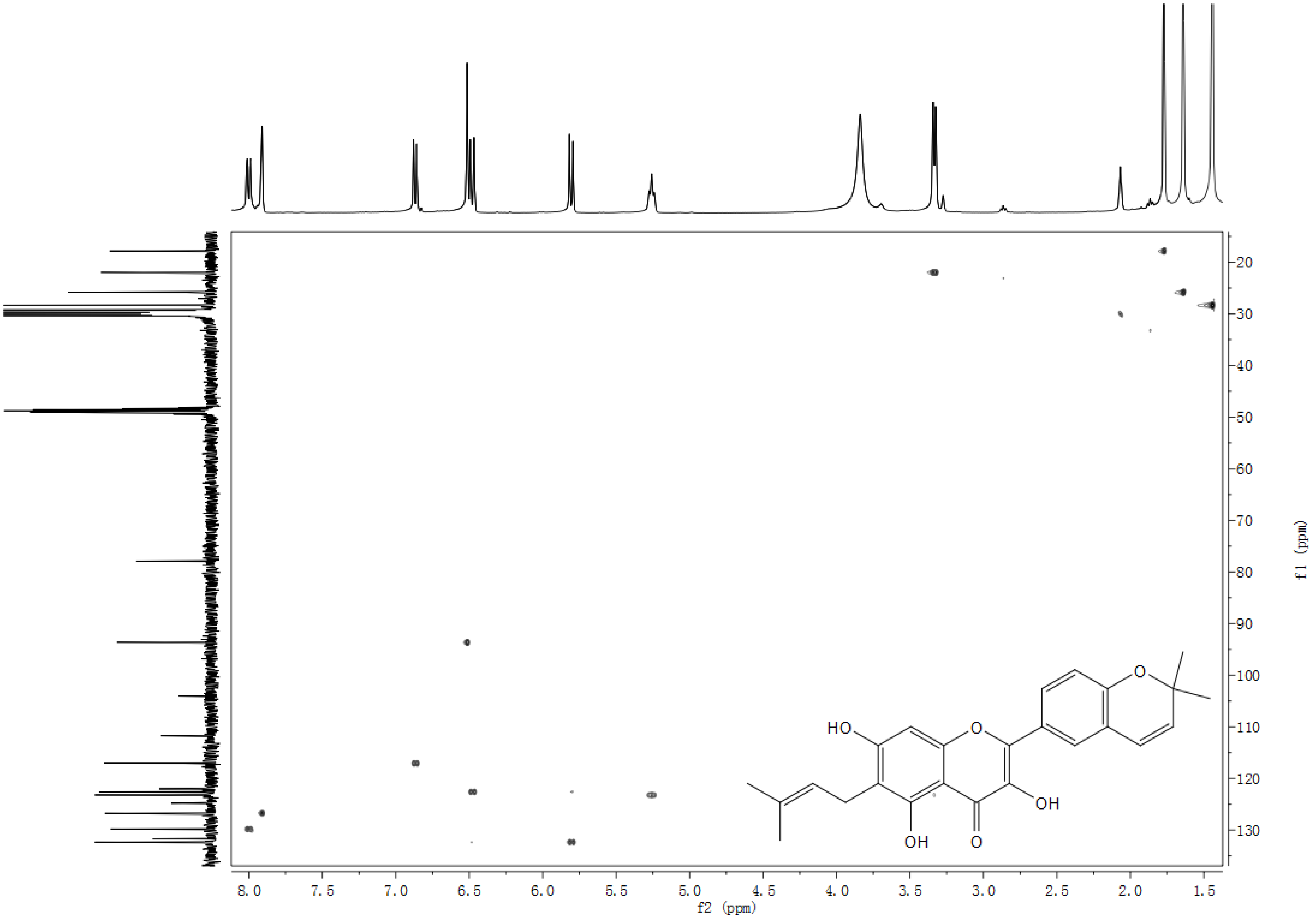

Figure S16. HSQC spectrum of Macadenanthin B (**2**)

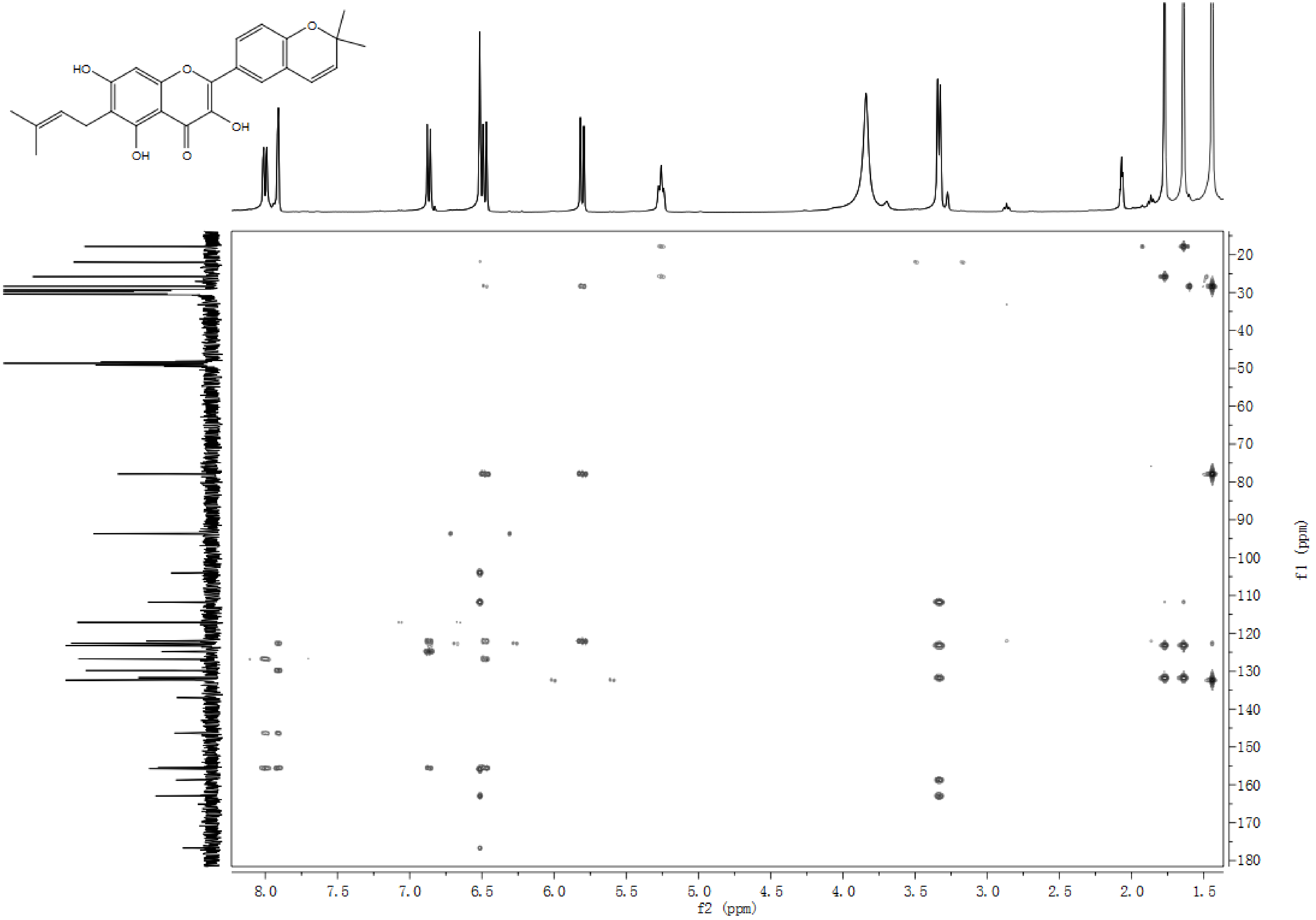

Figure S17. HMBC spectrum of Macadenanthin B (**2**)

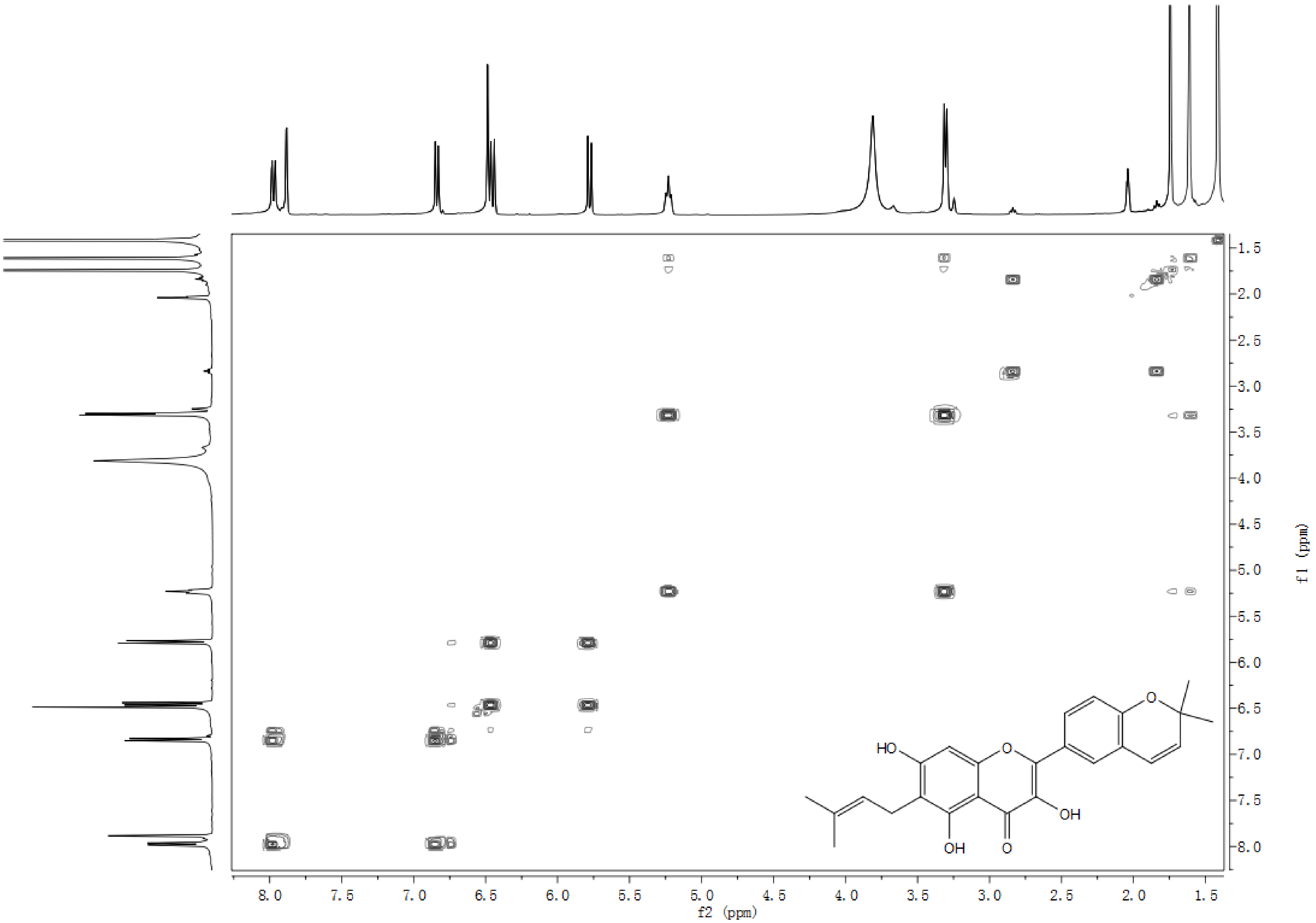

Figure S18. ^{1}H–^{1}H COSY spectrum of Macadenanthin B (**2**)

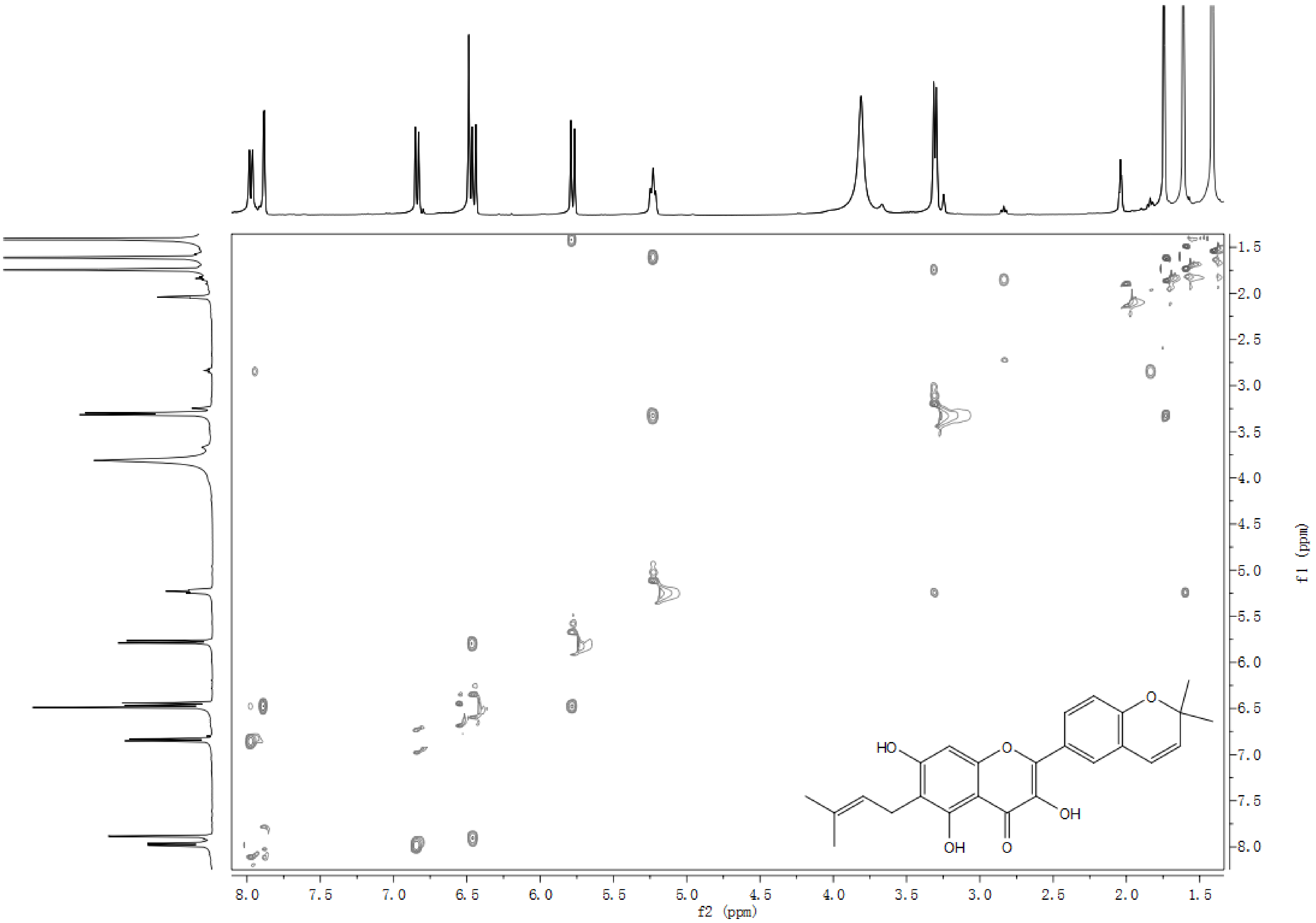

Figure S19. ROESY spectrum of Macadenanthin B (**2**)

Acquisition Parameter

Ion Source Type	ESI	Ion Polarity	Negative	Alternating Ion Polarity	off
Mass Range Mode	Ultra Scan	Scan Begin	100 m/z	Scan End	1200 m/z
Capillary Exit	-200.0 Volt	Skimmer	-40.0 Volt	Trap Drive	35.4
Accumulation Time	100000 µs	Averages	5 Spectra	Auto MS/MS	off

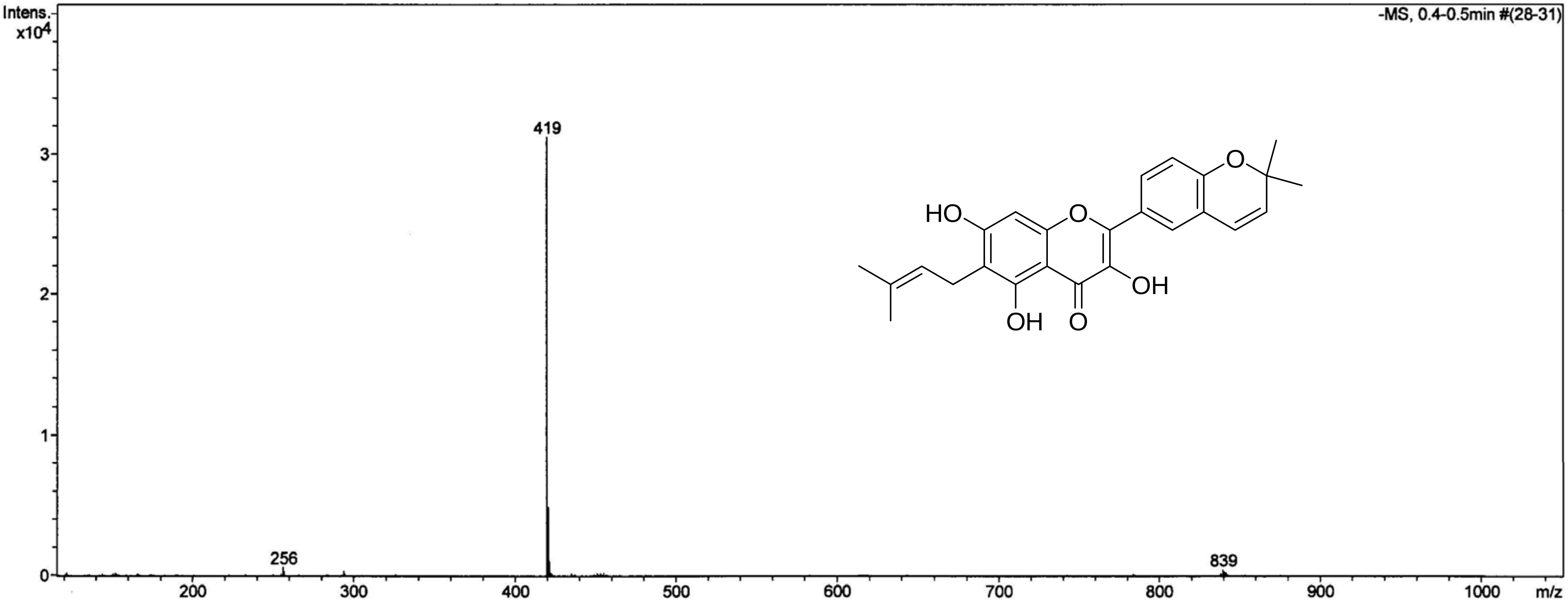

Figure S20. ESIMS of Macadenanthin B (**2**)

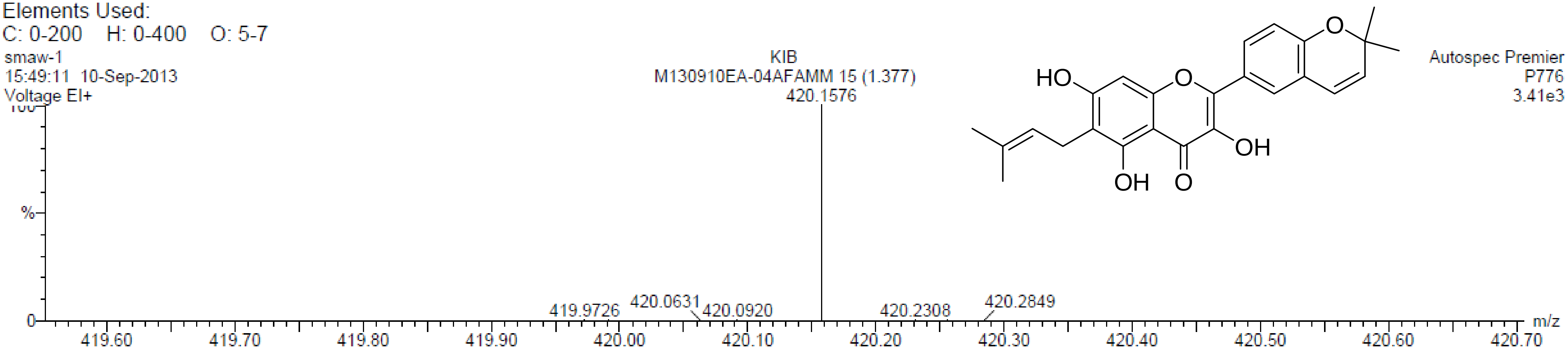

Minimum:				-10.0		
Maximum:		200.0	10.0	120.0		
Mass	Calc. Mass	mDa	PPM	DBE	i-FIT	Formula
420.1576	420.1573	0.3	0.7	14.0	5547722.5	C25 H24 O6

Figure S21. HREIMS of Macadenanthin B (**2**)

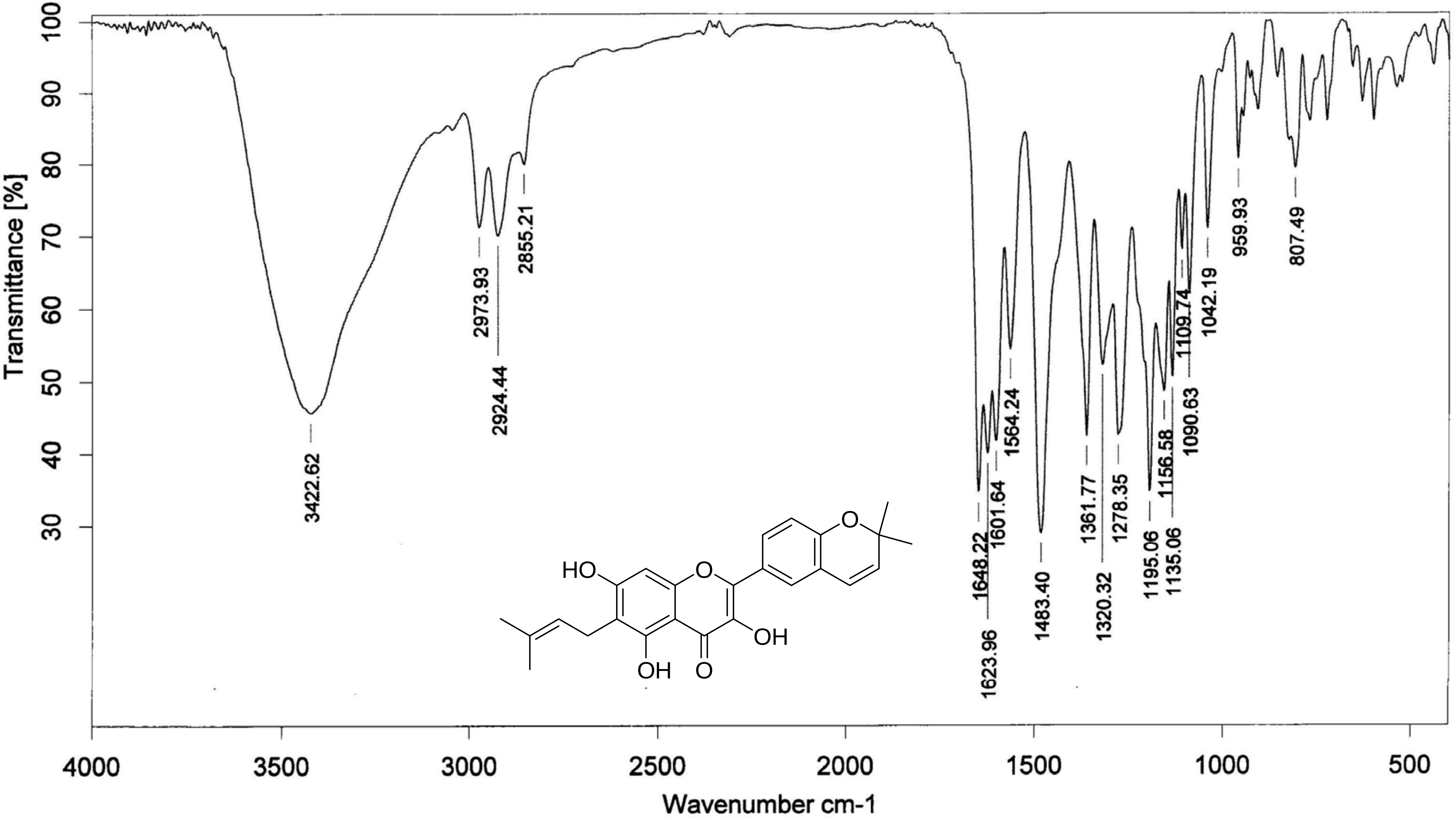

Figure S22. IR spectrum of Macadenanthin B (**2**)

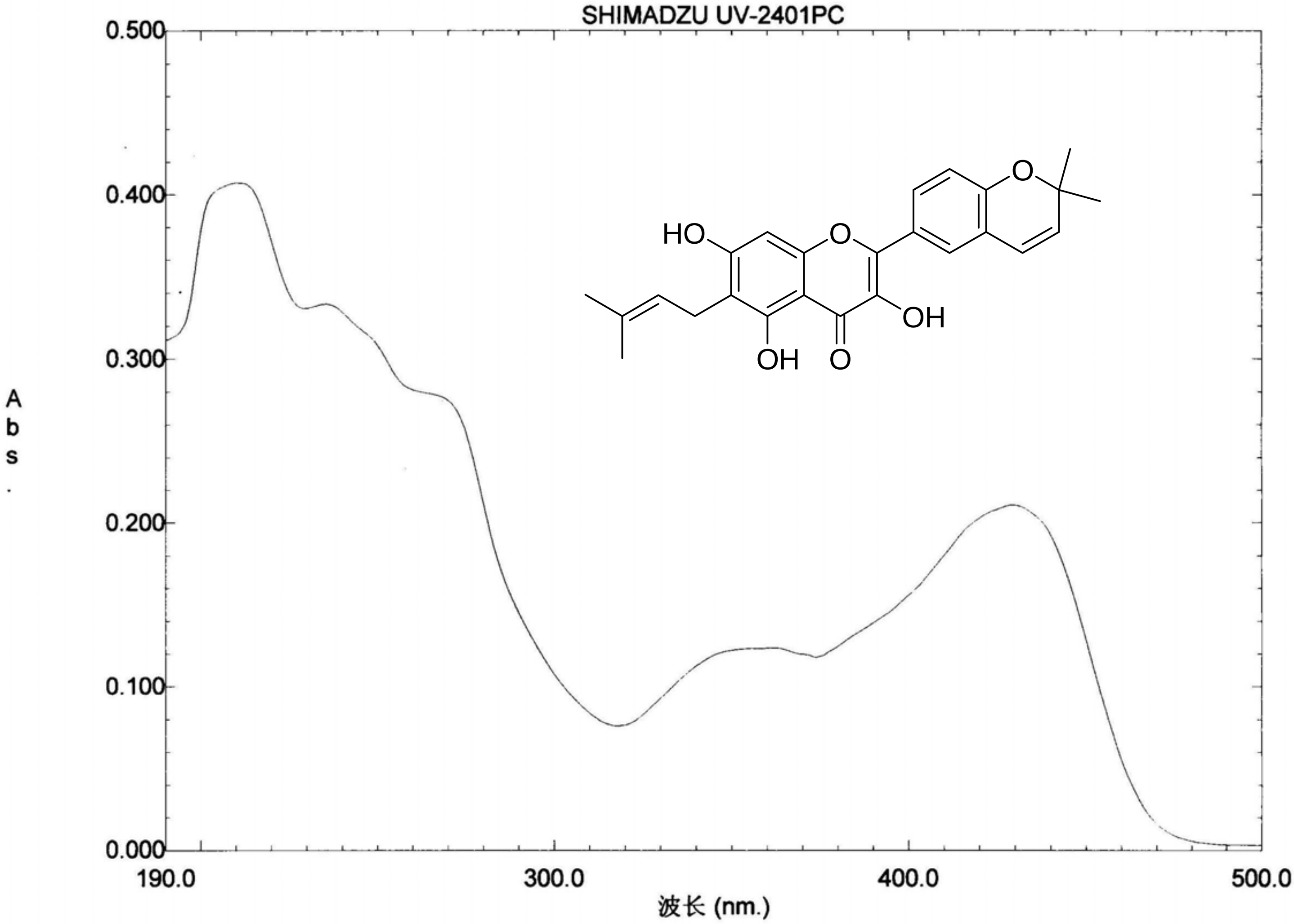

文件名:　SMAW-1

SMAW-1———

样品浓度：0.0054毫克/毫升
溶剂：甲醇

创建于:　19:42　13-09-10
数据:　原始

测量模式:　Abs.
扫描速度:　中速
狭缝:　5.0
采样间隔:　0.5

否.	波长 (nm.)	Abs.
1	552.50	0.0032
2	429.50	0.2107
3	361.00	0.1237
4	235.50	0.3329
5	211.00	0.4071

Figure S23. UV spectrum of Macadenanthin B (**2**)

Optical rotation measurement

Model : P-1020 (A060460638)

No.	Sample	Mode	Data	Monitor<br>Blank	Temp.<br>Cell<br>Temp Point	Date<br>Comment<br>Sample Name	Light<br>Filter<br>Operator	Cycle Time<br>Integ Time
No.1	3 (1/3)	Sp.Rot	-2.9500	-0.0010<br>0.0000	23.7<br>10.00<br>Cell	Tue Sep 10 18:25:55 2013<br>0.00339g/ml MeOH<br>SMAW-1	Na<br>589nm	2 sec<br>10 sec
No.2	3 (2/3)	Sp.Rot	-2.6550	-0.0009<br>0.0000	23.7<br>10.00<br>Cell	Tue Sep 10 18:26:09 2013<br>0.00339g/ml MeOH<br>SMAW-1	Na<br>589nm	2 sec<br>10 sec
No.3	3 (3/3)	Sp.Rot	-1.7700	-0.0006<br>0.0000	23.7<br>10.00<br>Cell	Tue Sep 10 18:26:22 2013<br>0.00339g/ml MeOH<br>SMAW-1	Na<br>589nm	2 sec<br>10 sec

-2.4583°

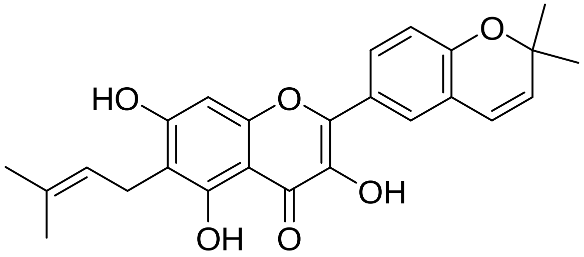

Figure S24. ORD spectrum of Macadenanthin B (**2**)

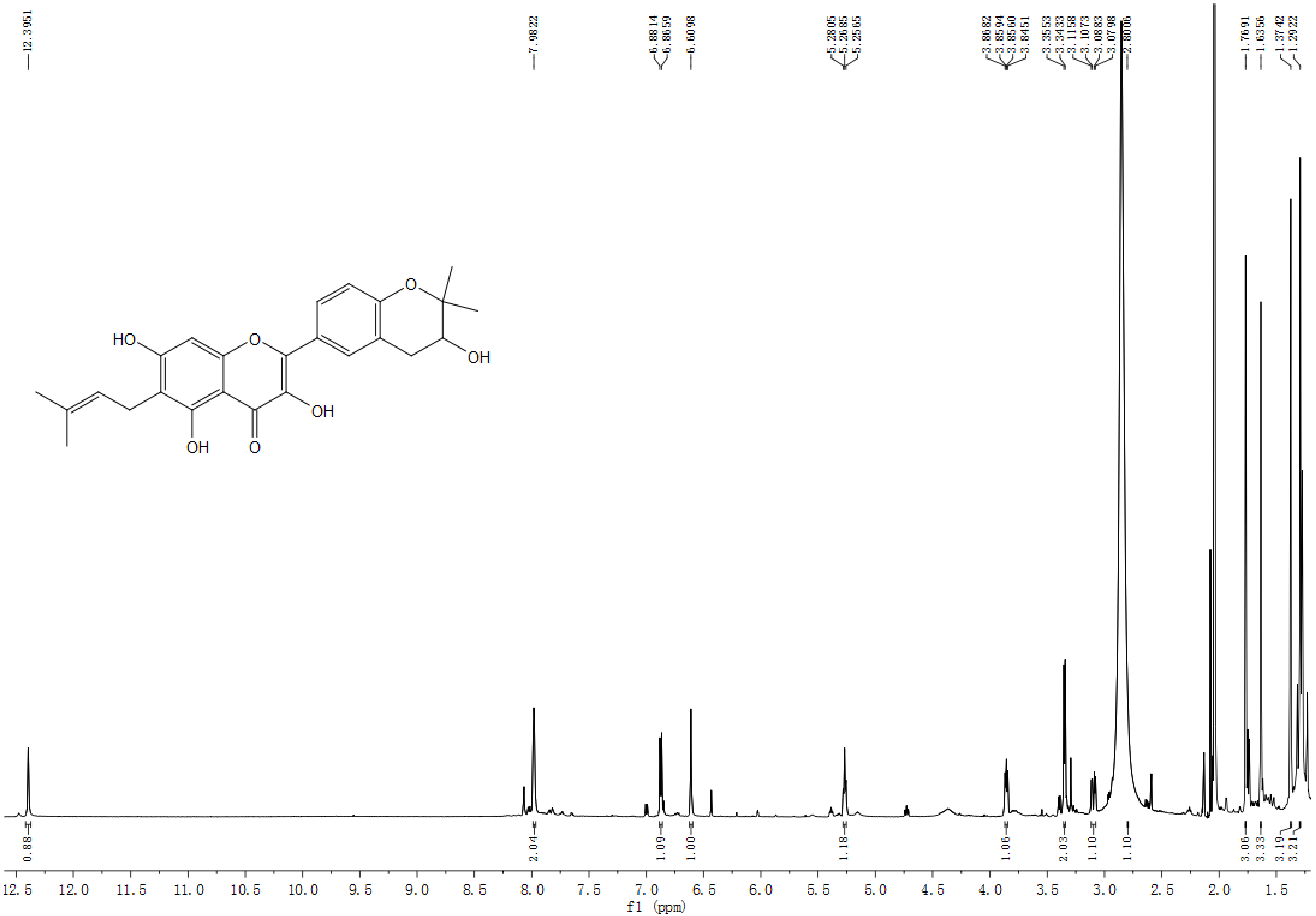

Figure S25. ^{1}H NMR spectrum of Macadenanthin C (**3**)

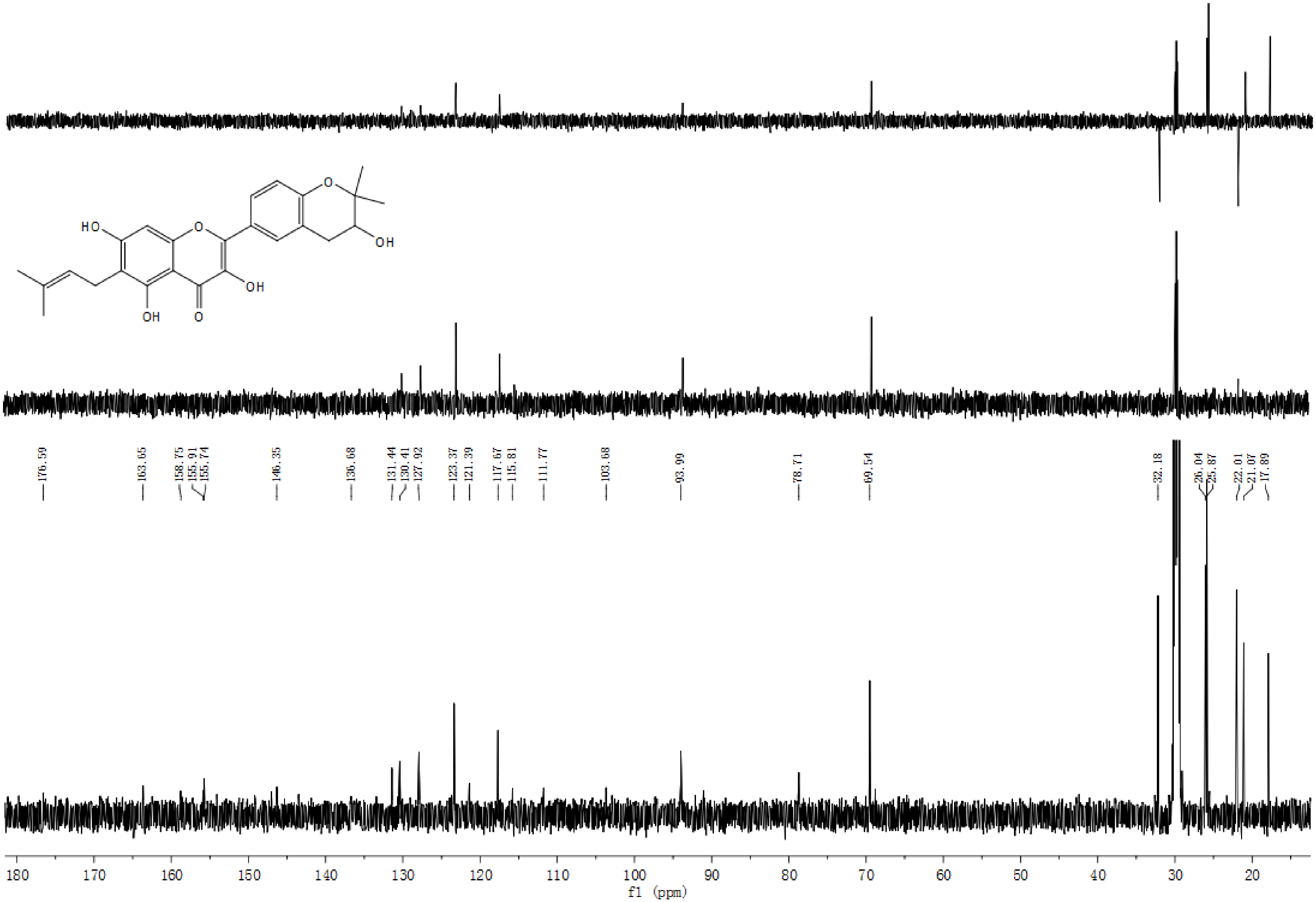

Figure S26. ^{13}C NMR spectrum of Macadenanthin C (**3**)

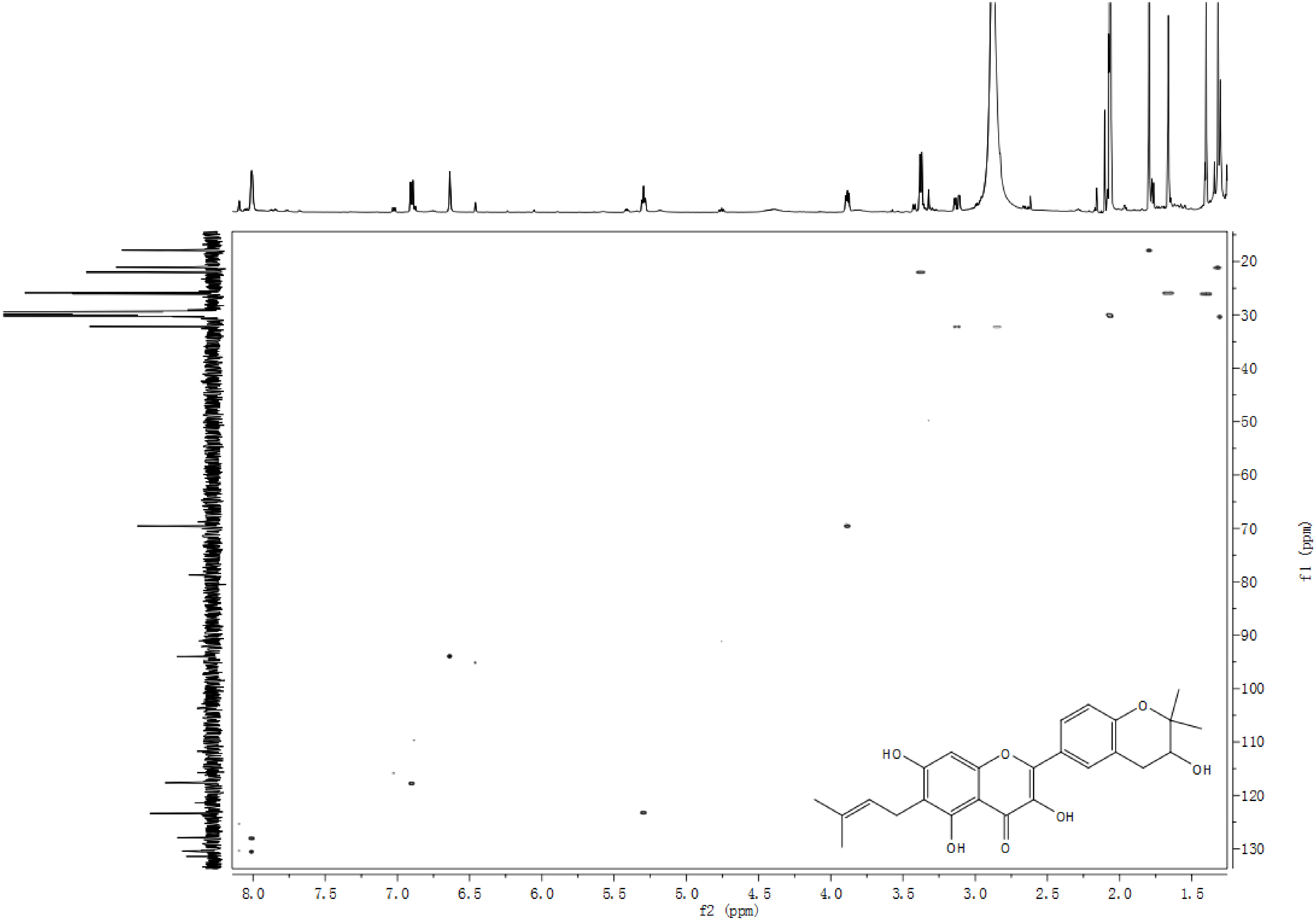

Figure S27. HSQC spectrum of Macadenanthin C (**3**)

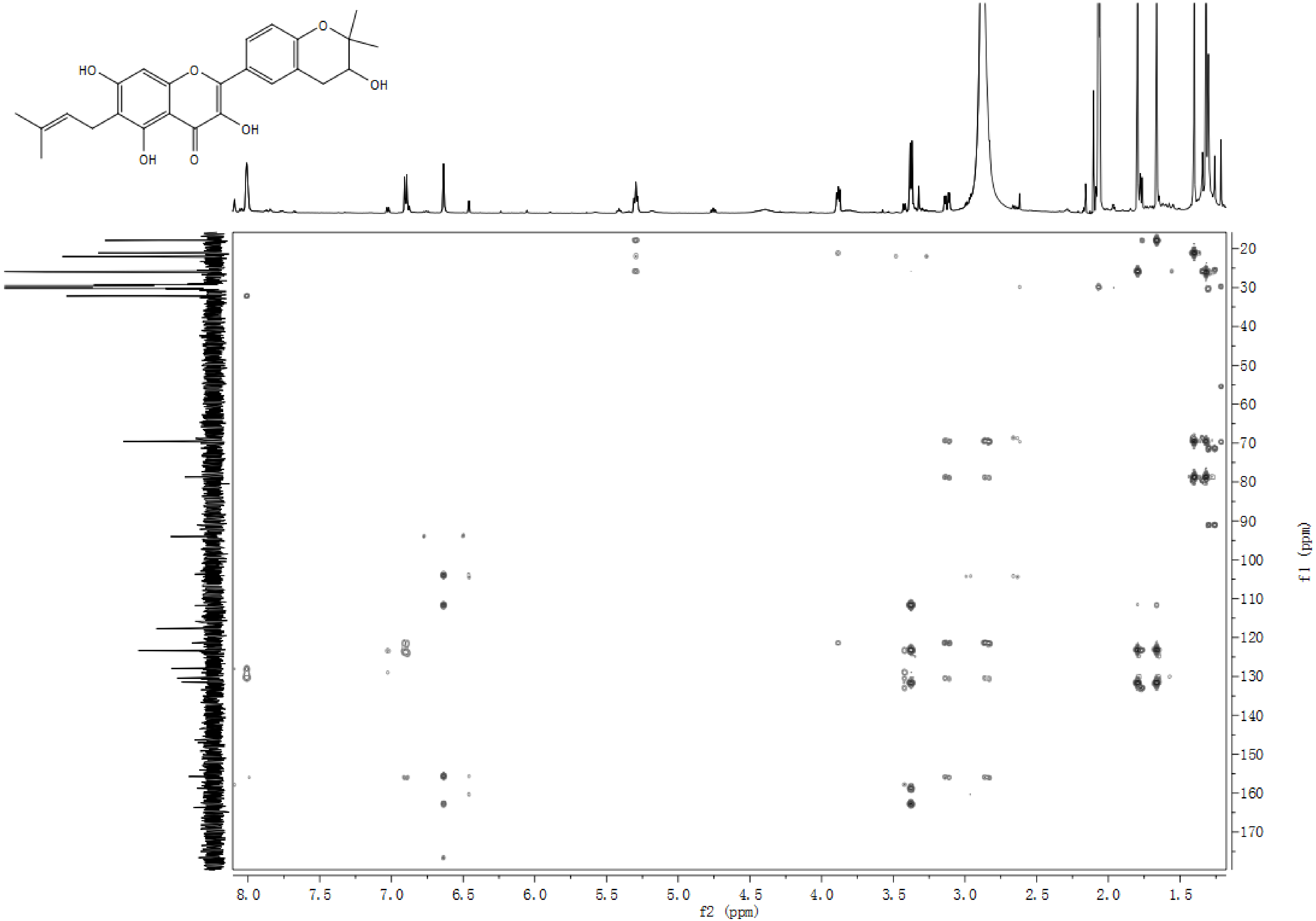

Figure S28. HMBC spectrum of Macadenanthin C (**3**)

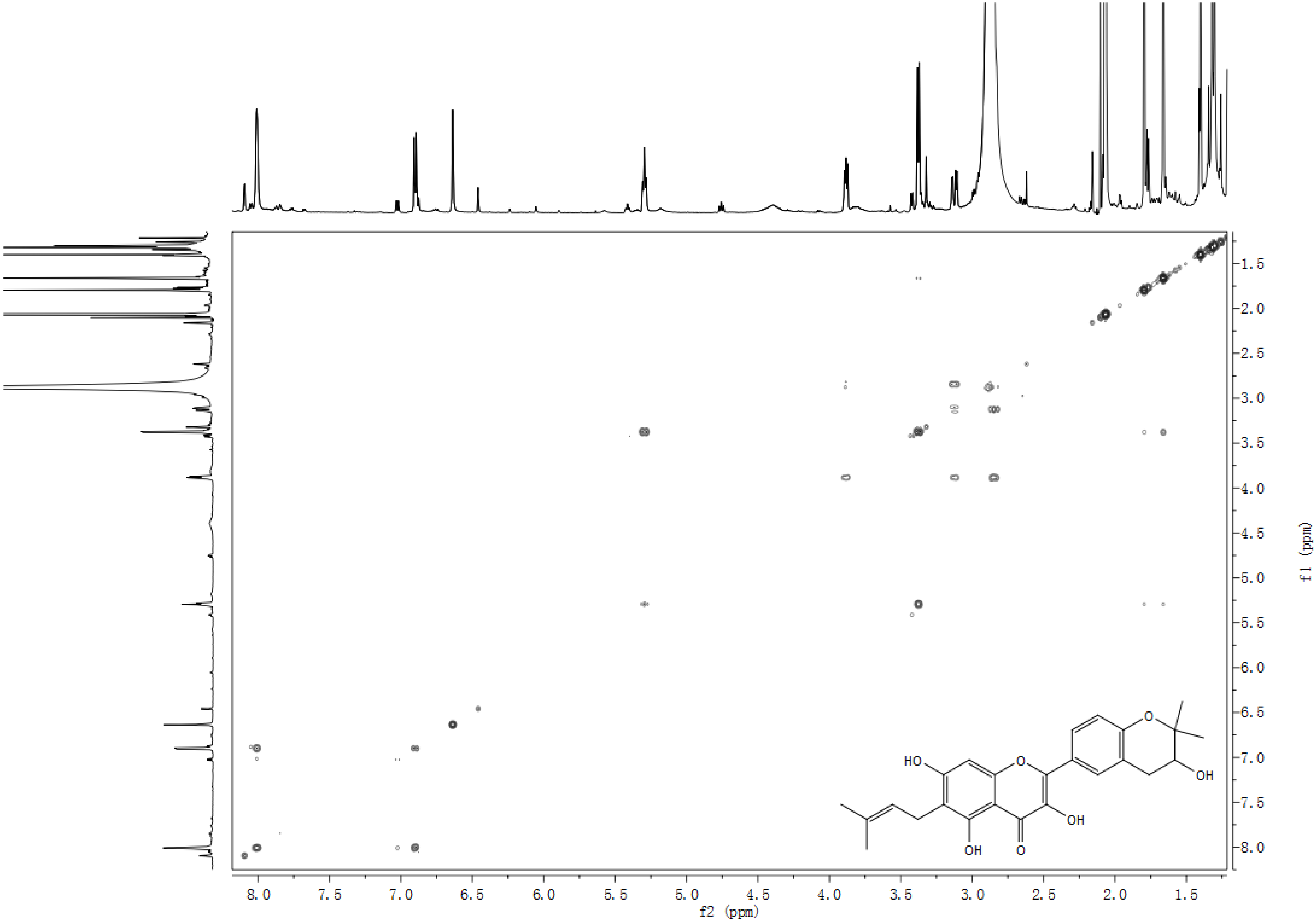

Figure S29. ^{1}H–^{1}H COSY spectrum of Macadenanthin C (**3**)

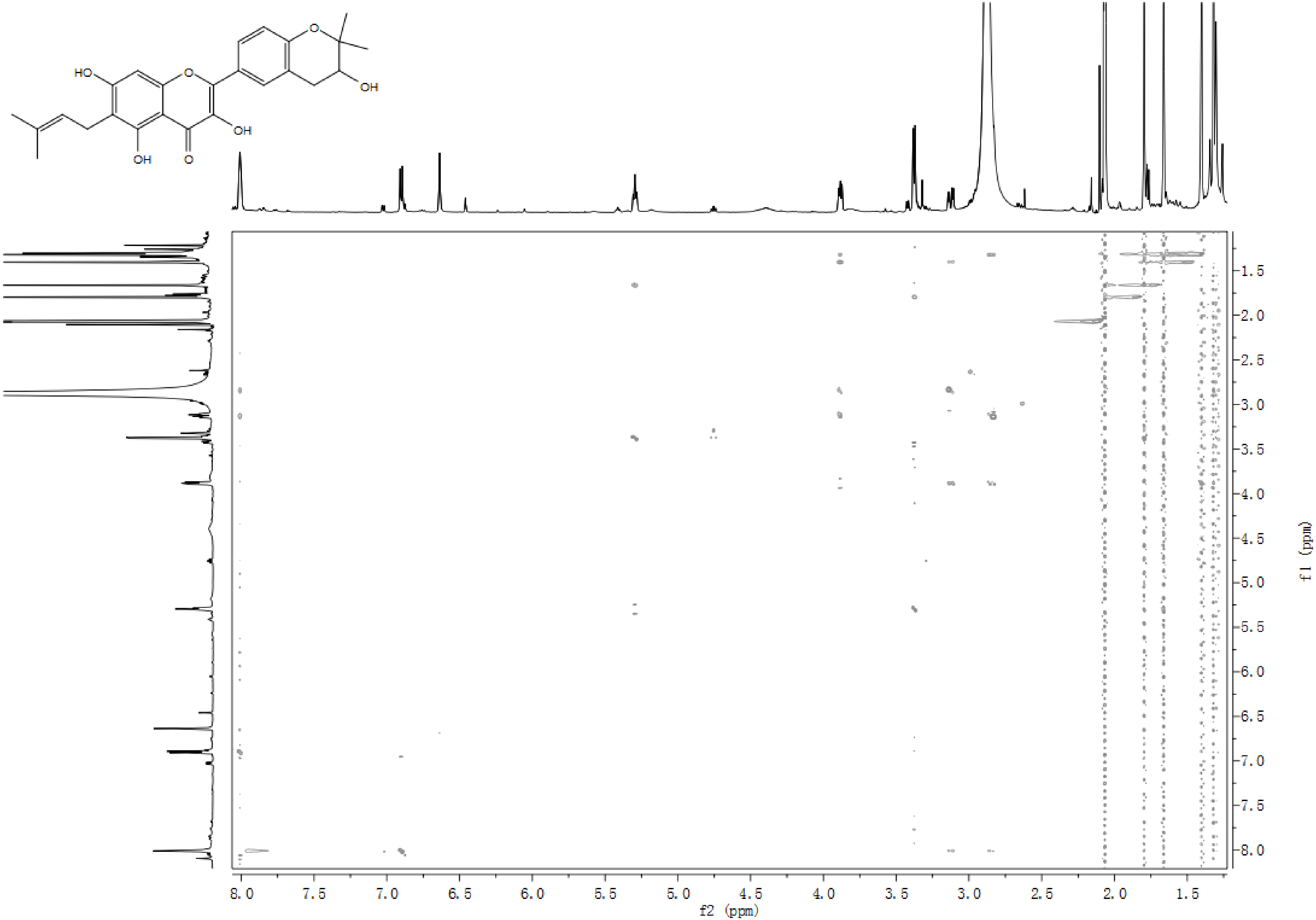

Figure S30. ROESY spectrum of Macadenanthin C (**3**)

Acquisition Parameter

Ion Source Type	ESI	Ion Polarity	Negative	Alternating Ion Polarity	off
Mass Range Mode	Ultra Scan	Scan Begin	100 m/z	Scan End	1200 m/z
Capillary Exit	-300.0 Volt	Skimmer	-40.0 Volt	Trap Drive	36.0
Accumulation Time	100000 µs	Averages	5 Spectra	Auto MS/MS	off

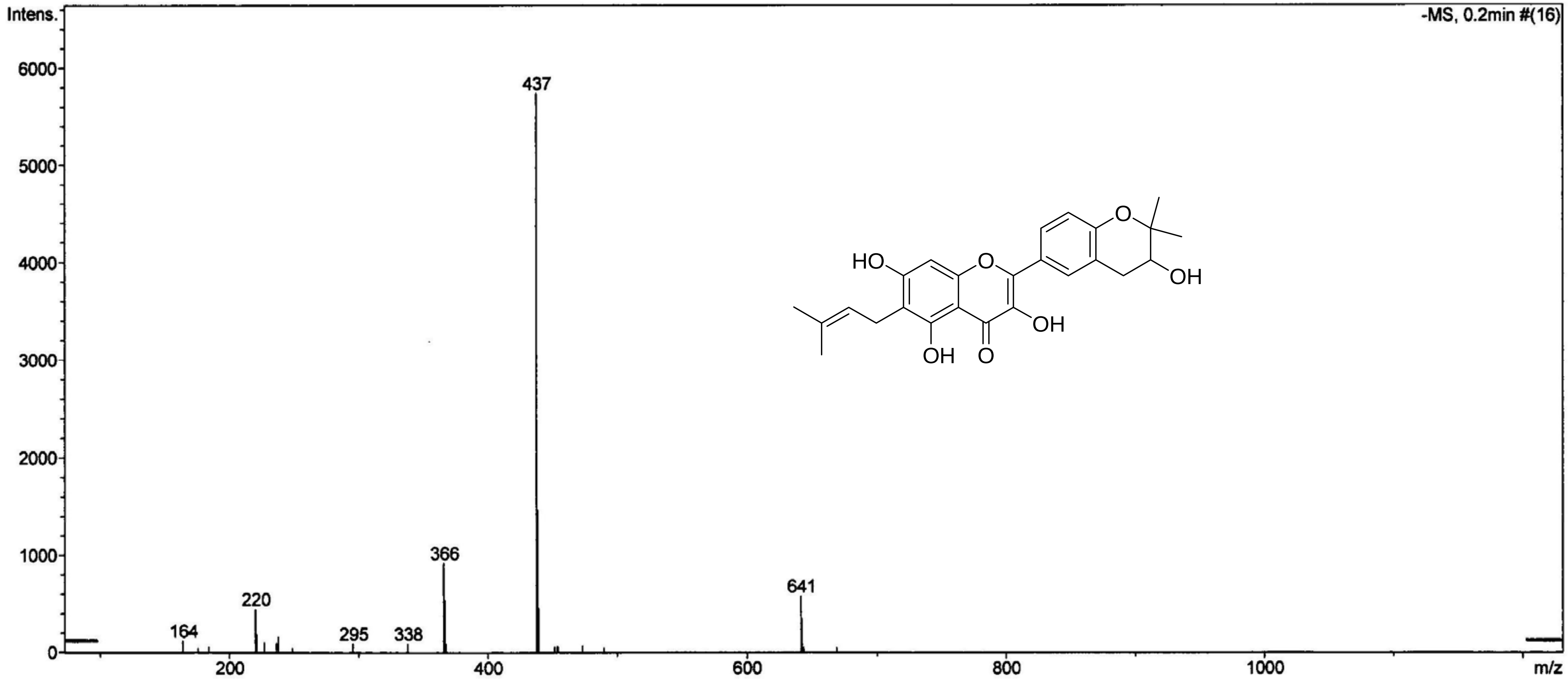

Figure S31. ESIMS of Macadenanthin C (**3**)

Elements Used:
C: 0-200   H: 0-400   O: 6-8

smaw-4a
10:36:46 19-Nov-2013
Voltage EI+

KIB
M131119EA-02AFAMM 20 (1.836)
438.1675

Autospec Premier
P776
307

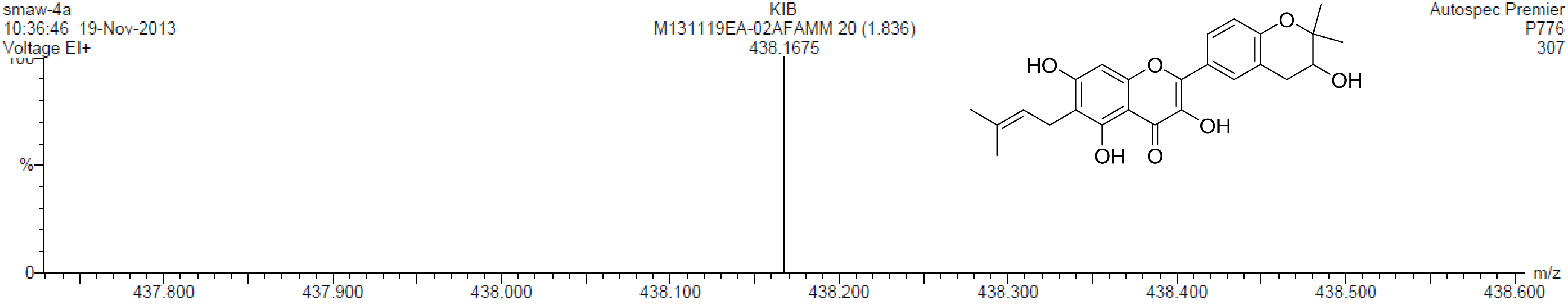

Minimum:				-10.0		
Maximum:		200.0	10.0	120.0		
Mass	Calc. Mass	mDa	PPM	DBE	i-FIT	Formula
438.1675	438.1679	-0.4	-0.9	13.0	5546169.0	C25 H26 O7

Figure S32. HREIMS of Macadenanthin C (**3**)

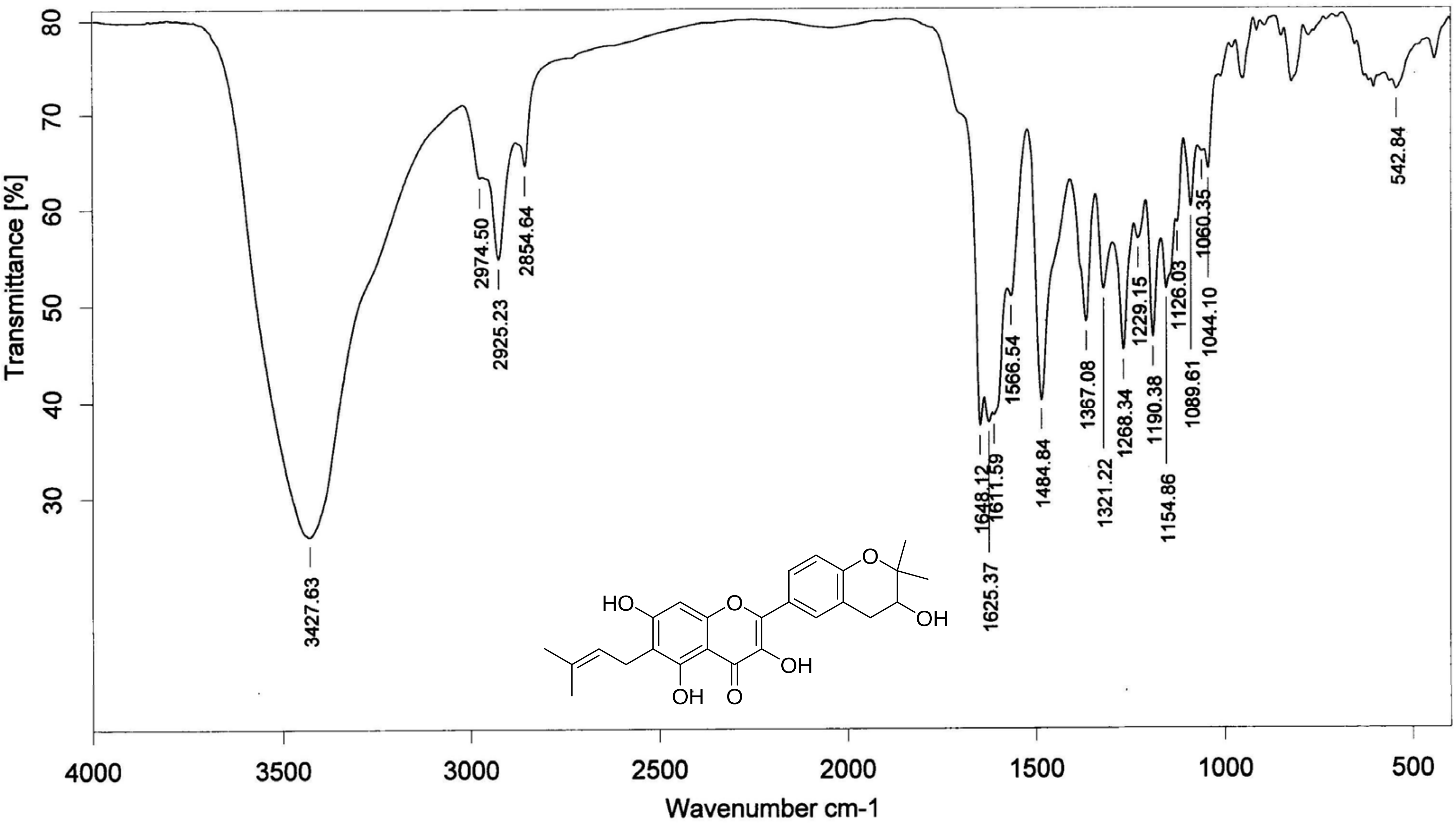

Figure S33. IR spectrum of Macadenanthin C (**3**)

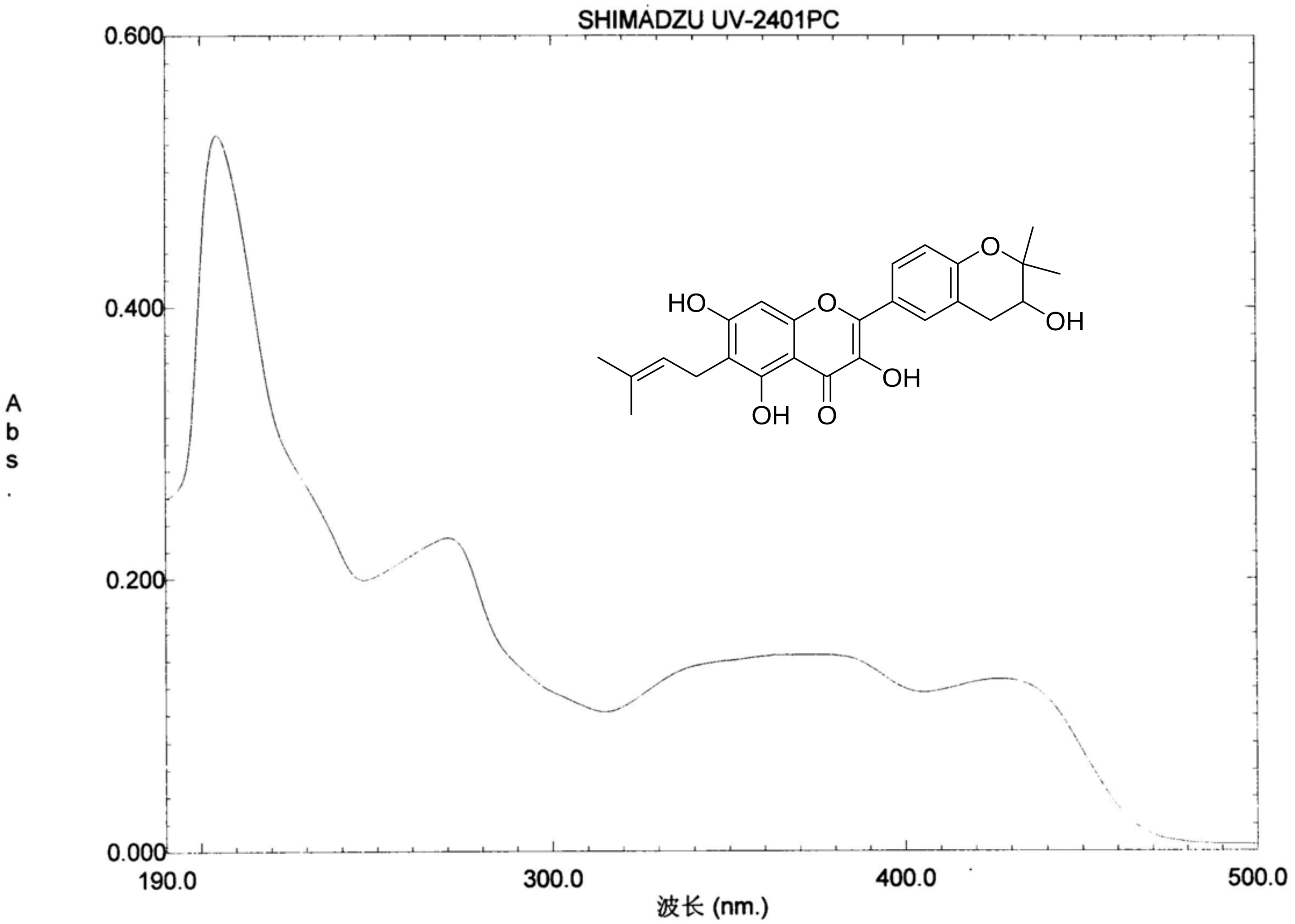

文件名: SMAW-4A

SMAW-4A

样品浓度：0.0034毫克/毫升
溶剂：甲醇

创建于: 16:27 13-11-22
数据: 原始

测量模式: Abs.
扫描速度: 中速
狭缝: 5.0
采样间隔: 0.5

否.	波长 (nm.)	Abs.
1	426.50	0.1259
2	376.00	0.1439
3	270.50	0.2303
4	204.50	0.5266

Figure S34. UV spectrum of Macadenanthin C (**3**)

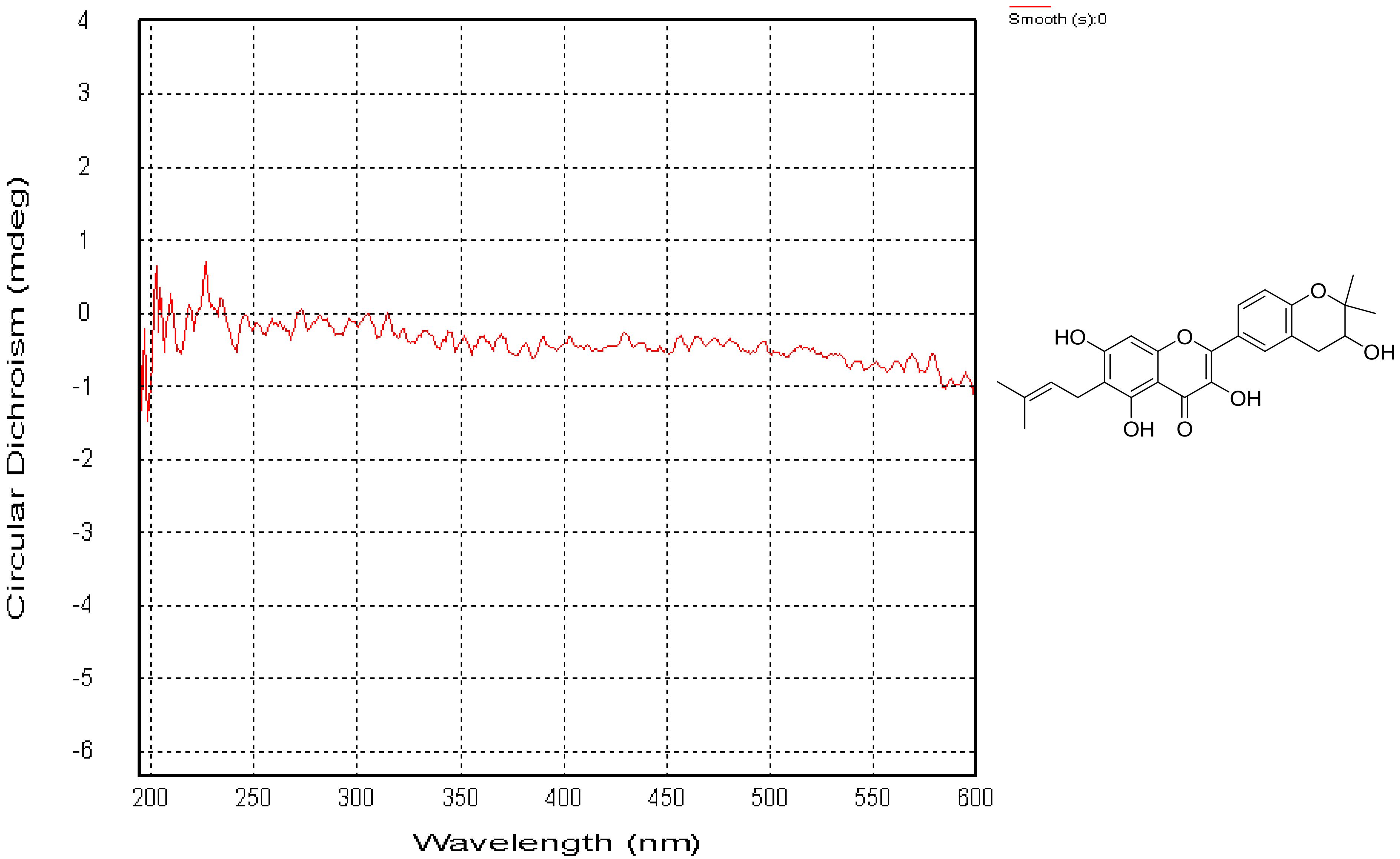

Figure S35. CD spectrum of Macadenanthin C (**3**)

Optical rotation measurement

Model : P-1020 (A060460638)

No.	Sample	Mode	Data	Monitor<br>Blank	Temp.<br>Cell<br>Temp Point	Date<br>Comment<br>Sample Name	Light<br>Filter<br>Operator	Cycle Time<br>Integ Time
No.1	12 (1/3)	Sp.Rot	-3.3060	-0.0004<br>0.0000	21.2<br>10.00<br>Cell	Fri Nov 22 15:16:02 2013<br>0.00121g/mL MeOH<br>SMAW-4A	Na<br>589nm	2 sec<br>10 sec
No.2	12 (2/3)	Sp.Rot	-3.3060	-0.0004<br>0.0000	21.2<br>10.00<br>Cell	Fri Nov 22 15:16:15 2013<br>0.00121g/mL MeOH<br>SMAW-4A	Na<br>589nm	2 sec<br>10 sec
No.3	12 (3/3)	Sp.Rot	-3.3060	-0.0004<br>0.0000	21.2<br>10.00<br>Cell	Fri Nov 22 15:16:29 2013<br>0.00121g/mL MeOH<br>SMAW-4A	Na<br>589nm	2 sec<br>10 sec

-3.3058°

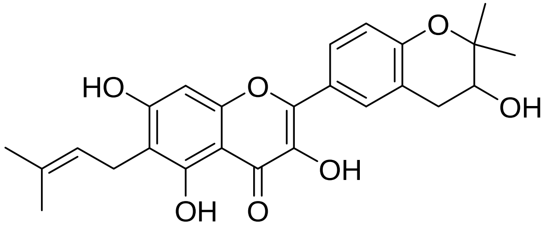

Figure S36. ORD spectrum of Macadenanthin C (**3**)
